# Structures Illuminate Cardiac Ion Channel Functions in Health and in Long QT Syndrome

**DOI:** 10.3389/fphar.2020.00550

**Published:** 2020-05-04

**Authors:** Kathryn R. Brewer, Georg Kuenze, Carlos G. Vanoye, Alfred L. George, Jens Meiler, Charles R. Sanders

**Affiliations:** ^1^Center for Structural Biology, Vanderbilt University School of Medicine Basic Sciences, Nashville, TN, United States; ^2^Department of Biochemistry, Vanderbilt University, Nashville, TN, United States; ^3^Department of Chemistry, Vanderbilt University, Nashville, TN, United States; ^4^Department of Pharmacology, Feinberg School of Medicine, Northwestern University, Chicago, IL, United States; ^5^Department of Pharmacology, Vanderbilt University School of Medicine Basic Sciences, Nashville, TN, United States; ^6^Institute for Drug Discovery, Leipzig University Medical School, Leipzig, Germany

**Keywords:** cardiac action potential, long QT syndrome, KCNQ1, hERG, SCN5A, structural biology

## Abstract

The cardiac action potential is critical to the production of a synchronized heartbeat. This electrical impulse is governed by the intricate activity of cardiac ion channels, among them the cardiac voltage-gated potassium (K_v_) channels KCNQ1 and hERG as well as the voltage-gated sodium (Na_v_) channel encoded by *SCN5A*. Each channel performs a highly distinct function, despite sharing a common topology and structural components. These three channels are also the primary proteins mutated in congenital long QT syndrome (LQTS), a genetic condition that predisposes to cardiac arrhythmia and sudden cardiac death due to impaired repolarization of the action potential and has a particular proclivity for reentrant ventricular arrhythmias. Recent cryo-electron microscopy structures of human KCNQ1 and hERG, along with the rat homolog of SCN5A and other mammalian sodium channels, provide atomic-level insight into the structure and function of these proteins that advance our understanding of their distinct functions in the cardiac action potential, as well as the molecular basis of LQTS. In this review, the gating, regulation, LQTS mechanisms, and pharmacological properties of KCNQ1, hERG, and SCN5A are discussed in light of these recent structural findings.

## Introduction

The cardiac action potential is critical to proper heart function. Beginning with the activation of “pacemaker” cells, the action potential propagates through the atria and into the ventricles in a unidirectional waveform of excitation and relaxation, resulting in the coordinated expansion and contraction of heart tissue (Nerbonne and Kass, 2005). The action potential is governed by an intricate series of ion channel activities (Grant, 2009), including those of the KCNQ1 (K_V_LQT1, K_V_7.1) and hERG (KCNH2, K_V_11.1) potassium channels and the SCN5A (Na_V_1.5) sodium channel. Mutations in these three channels are the most frequent cause of congenital long QT syndrome (LQTS), a cardiac arrhythmia disorder that is one of the primary causes of sudden arrhythmic death syndrome (SADS) (Skinner et al., 2019).

**Figure 1:**
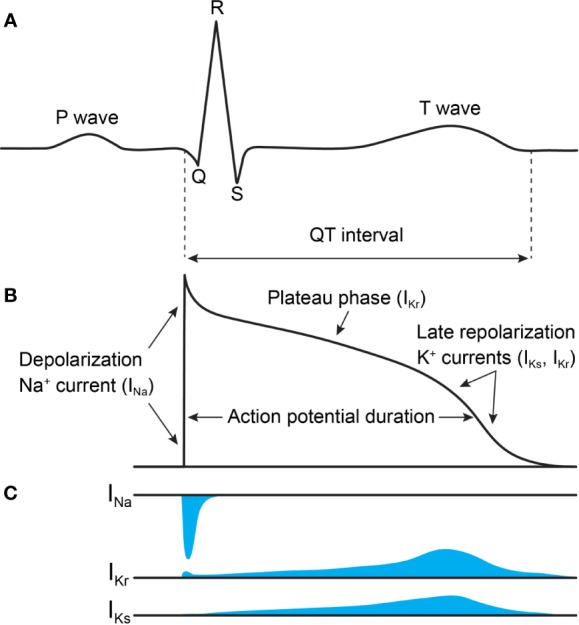
Voltage-activated Na^+^ and K^+^ currents define the ventricular action potential and QT interval of the ECG. **(A)** ECG trace. The rapid upstroke of the ventricular action potential gives rise to the QRS complex. The duration of the QT interval is determined by the time of the ventricular repolarization. **(B)** Trace of the ventricular action potential. The rapidly activating and inactivating I_Na_ current drives membrane depolarization. Two K^+^ currents, I_Ks_ and I_Kr_, contribute most to the plateau phase and repolarization phase of the action potential, which reestablishes the membrane resting potential. **(C)** Time course of I_Na_, I_Kr_, and I_Ks_ currents (not drawn to scale). Currents of other ion channels contributing to the action potential (e.g. I_Ca,L_, I_Kur_, and I_NCX_) are not shown for clarity. This figure is inspired by figures in Moss and Kass (2005) and George (2013).

KCNQ1, hERG, and SCN5A each play a distinct role in generating the cardiac action potential (**Figure 1**), consequently producing distinct LQTS forms when mutated (Skinner et al., 2019). The initial upstroke is governed primarily by SCN5A, producing the I_Na_ current that amplifies membrane depolarization and propagates the action potential (Moss and Kass, 2005; Skinner et al., 2019). Mutation in SCN5A causes LQTS type 3 (LQT3). hERG shapes both the plateau and the repolarization phases of the action potential, along with KCNQ1 in complex with the KCNE1 accessory protein (Moss and Kass, 2005; George, 2013). hERG and the KCNQ1–KCNE1 complex produce the rapid (I_Kr_) and slow (I_Ks_) delayed rectifier currents (**Figures 1B, C**), respectively. KCNQ1 mutation causes LQT1, and hERG, LQT2 (Skinner et al., 2019). Additional ion channels and transporters also shape the cardiac action potential (Skinner et al., 2019), among them Ca_v_1.2, NCX1, and Kir2.1. The roles of these channels in the cardiac action potential and cardiac arrhythmias have been reviewed elsewhere (Bidaud and Lory, 2011; Hancox et al., 2018; Meza et al., 2018; Vaidyanathan et al., 2018; Li et al., 2019). These channels are not typically causative for LQTS and will not be discussed here.

**Figure 2:**
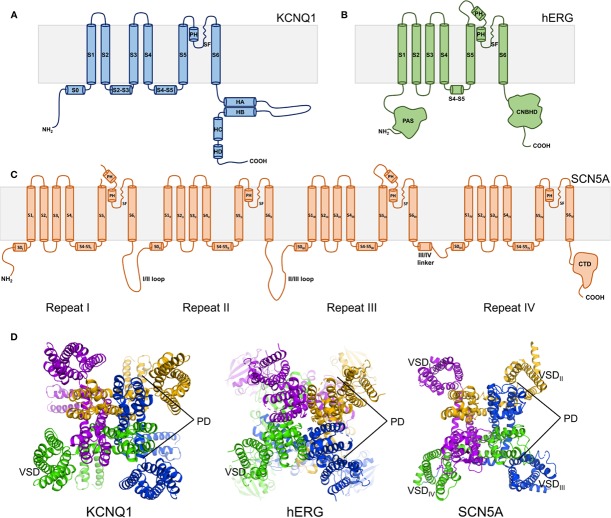
Overall topology of KCNQ1, hERG, and SCN5A channels. **(A)** KCNQ1 topology. Transmembrane domain alpha helices are labeled S0–S6. PH indicates the pore helix. SF denotes the selectivity filter. S2–S3 indicates the S2–S3 linker. S4–S5 denotes the S4–S5 linker. Cytosolic alpha helices are labeled HA-HD. **(B)** hERG topology. PAS denotes the Per-ARNT-Sim domain. CNBHD indicates the C-terminal cyclic nucleotide-binding homology domain. **(C)** SCN5A topology. CTD indicates the C-terminal domain. **(D)** Top views of the human KCNQ1 (PDB ID: 6UZZ) (Sun and Mackinnon, 2020), human hERG (PDB ID: 5VA1) (Wang and Mackinnon, 2017), and rat SCN5A (PDB ID: 6UZ3) (Jiang et al., 2020) channels, respectively. Outer voltage-sensing domains (VSD) and the central pore domain (PD) are labeled.

Structurally, the KCNQ1, hERG, and SCN5A channels belong to the voltage-gated ion channel superfamily and share a general transmembrane topology (Bezanilla, 2005; Dehghani-Samani et al., 2019). The transmembrane channel domain is composed of six helices per subunit in hERG and KCNQ1 (**Figures 2A, B**) or a monomeric tetrad repeat of linked 6-helix domains for SCN5A, each repeat exhibiting varying sequences, lengths, and tertiary folds (**Figures 2C, D**). The assembled channel is tetrameric (pseudo-tetrameric for SCN5A), with each channel domain composed of four voltage sensing domains (VSDs) surrounding a central pore domain (PD) (**Figure 2D**). The VSD is comprised of the first four transmembrane helices (S1–S4) preceded by a small amphipathic helix—S0—in both KCNQ1 and SCN5A (Jiang et al., 2020; Sun and Mackinnon, 2020). The PD is formed by the tetramerization of S5 and S6 helices from each subunit/repeat (**Figure 2D**). A pore loop between S5 and S6 contains the selectivity filter (SF) that confers ion specificity. A linker helix between helices S4 and S5, termed the S4–S5 linker, connects the VSD to the PD (Bezanilla, 2005; Dehghani-Samani et al., 2019) (**Figures 2A–C**).

Given these similarities in channel topology and components, how is it that KCNQ1, hERG, and SCN5A perform such distinct functions, and produce phenotypically distinct forms of LQTS? To explore this question, a number of high-resolution ion channel structures, including cryo-electron microscopy (cryo-EM) structures of frog and human KCNQ1 (Sun and Mackinnon, 2020) and of human hERG (Wang and Mackinnon, 2017), as well as the structures of the rat homolog of SCN5A (Jiang et al., 2020) and human Na_v_1 isoforms Nav1.4 and 1.7 (Pan et al., 2018; Shen et al., 2019; Xu et al., 2019), have been determined. Moreover, the frog KCNQ1 structure has been used to develop what is likely a reliable homology model for the human KCNQ1 channel in resting and fully active conformations (Kuenze et al., 2019). A homology model of human SCN5A in the resting state has also been devised (Kroncke et al., 2019). These structures and structural models reveal critical differences in the atomic details of KCNQ1, hERG, and SCN5A structures associated with their distinct functions and disease phenotypes. Notably, the subunits of KCNQ1 undergo domain swapping, with a similar arrangement observed in SCN5A but not in the hERG channel (**Figure 2D**). The monomeric sequence of SCN5A causes the channel to adopt an asymmetric three-dimensional fold, in contrast to the inherent symmetry of tetrameric hERG and KCNQ1 (**Figure 2D**). Additionally, the C-terminal domains contain distinct folds and mediate unique regulatory functions. These and other structural differences contribute to the varying properties of these three channels and to their distinct roles in the cardiac action potential.

The aim of this review is to compare and contrast the KCNQ1, hERG, and SCN5A channels using available structures and structural models as a guide. Through this lens, channel gating, regulation, LQTS mechanisms, and pharmacology will be discussed, in order to explore the molecular basis of these unique properties.

## Structural Mechanisms of Channel Gating

KCNQ1, hERG, and SCN5A undergo conformational changes in response to changes in membrane potential that result in channel opening or closing. These responses confer specific gating properties including activation, deactivation, inactivation, and recovery from inactivation (Hosseini, 2018; Zhang et al., 2018). In activation, protein conformational changes result in channel pore opening from a resting state, while deactivation entails a return to the resting state (Zhang et al., 2018). Inactivation confers a third channel state distinct from the activated and resting states which inhibits current flow prior to full deactivation (Zhang et al., 2018). While all three channels share common structural elements that are responsible for producing these states, there are also elements that give rise to specific gating properties in each channel, as discussed below.

### Activation and Ion Conduction

#### KCNQ1

**Figure 3:**
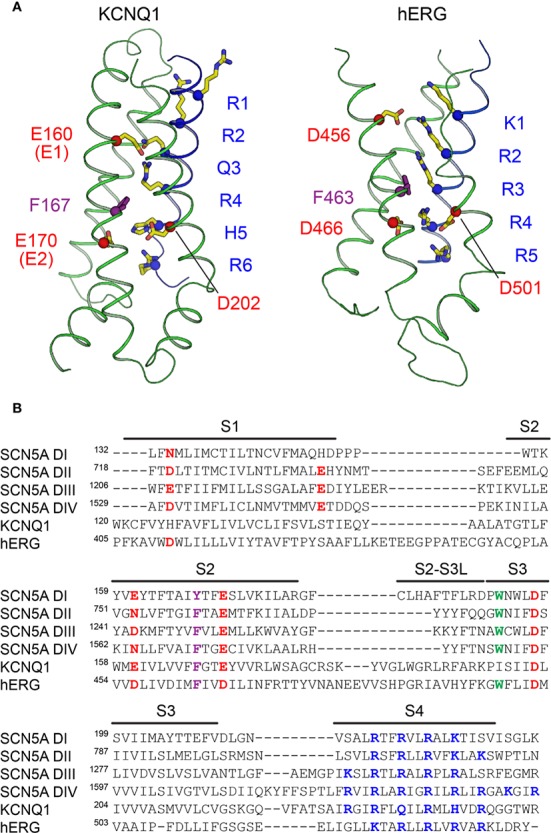
The voltage sensors of KCNQ1, hERG, and SCN5A. **(A)** Structure of the VSD from human KCNQ1 (left) (PDB: 6UZZ) (Sun and Mackinnon, 2020) and hERG (right) (PDB: 5VA2) (Wang and Mackinnon, 2017) in putative activated conformations. Basic residues on S4 (labeled in blue), acidic residues on S2 and S3 (labeled in red), and the phenylalanine residue in the gating charge transfer center (purple) are shown as sticks. The first four basic residues on S4 (R1–R4) in KCNQ1 and the first three (K1–R3) in hERG are located above the charge transfer center. **(B)** Multiple sequence alignment of VSDs I-IV of SCN5A with the VSDs of human KCNQ1 and hERG. Basic residues in S4 implicated with voltage sensing are colored blue, and acidic or polar residues in S1–S3 suggested to interact with S4 gating charges are colored red. The conserved aromatic residue at the gating charge transfer center in S2 is colored purple. Another tryptophan residue in S3, conserved in the VSDs of Na_V_ channels and also present in hERG, is colored green.

Voltage-gated channels contain up to six positively-charged basic residues in the S4 helix, called gating charges, which move in the electric field of the membrane in response to voltage (Jiang et al., 2003). Gating charges are numbered according to their position in the S4 helix, from the extracellular to the intracellular side. In KCNQ1, S4 contains four arginine (R) gating charges that are conserved in other K_V_ channels and confer voltage sensitivity. However, in KCNQ1 the canonical R3 is replaced by a neutral glutamine (Q234, Q3), and the fifth gating charge (K5 in Shaker) is replaced by histidine (H241, H5), which in the membrane environment is expected to be neutral at physiological pH (**Figures 3A, B**). Due to these substitutions at positions 3 and 5, the S4 helix of KCNQ1 has a lower net positive charge (+4) than Shaker-class K^+^ channels, such as K_V_1.2 (+6). The lower net positive charge may explain why KCNQ1 S4 mutations that result charge loss or reversal (Panaghie and Abbott, 2007; Wu et al., 2010b) result in constitutive channel activity.

Voltage-gated channels also feature a charge transfer center (CTC), formed by a bulky aromatic ring and two negatively charged residues, that facilitates S4 movement (Tao et al., 2010). The KCNQ1 CTC consists of E170 (E2) and F167 on S2, and D202 on S3, which work with E160 (E1) to define the S4 position. During activation, S4 moves towards the extracellular side of the membrane (Nakajo and Kubo, 2007; Rocheleau and Kobertz, 2008; Osteen et al., 2010; Ruscic et al., 2013; Barro-Soria et al., 2014; Nakajo and Kubo, 2014; Barro-Soria et al., 2017) through interactions between basic gating charges and E1 and E2 in the CTC (**Figure 3A**). These interactions change during the course of activation, permitting S4 translocation. E1 interacts with R1 (R228) or R4 (R237) in the resting and activated states of the VSD, respectively (Wu et al., 2010a). S4 motion occurs in two distinct steps, transitioning through a stable intermediate before reaching the activated state (Wu et al., 2010a; Barro-Soria et al., 2014; Zaydman et al., 2014). The intermediate state features salt bridge interactions between E1 and R2, distinct from the resting and activated VSD states, according to the recently-determined structure of the intermediate state KCNQ1 VSD (Taylor et al., 2020). Interestingly, the pore of KCNQ1 opens in both the intermediate state (IO) and fully activated (AO) states (Zaydman et al., 2014). These two open states possess distinct channel properties with differing opening probabilities and pharmacology (Hou et al., 2017), and have distinct pore structures (Zaydman et al., 2014). Importantly, ion conductance when the VSD is in either the intermediate or activated state appears to be unique to KCNQ1. However, formation of the KCNQ1–KCNE1 complex eliminates the conductance associated with the VSD intermediate state (Zaydman et al., 2014), such that I_Ks_ reflects only the fully activated state.

The ion conduction pathway in KCNQ1 is lined by the four S6 helices, with the SF on the extracellular side of the pore. Mutations in S6 cause changes in current amplitude and voltage dependence of activation (Wang et al., 1999; Seebohm et al., 2005; Panaghie et al., 2006; Hoosien et al., 2013). Comparison of S6 in the closed pore structure of human KCNQ1 (Sun and Mackinnon, 2020) with that of the open channel demonstrates that channel opening results from bending of S6 so that the cytosolic ends of the four S6 segments swing away from the central axis, enlarging the diameter of the pore to allow diffusion of K^+^ into the central cavity. The hinge responsible for this bending motion in S6 is the P343–A344–G345 (PAG) motif, which corresponds to PVP in Shaker K^+^ channels (Labro and Snyders, 2012). Additionally, A336 may also be important in the motion of the activation gate, as mutations at this position alter the voltage dependence of activation (Seebohm et al., 2006).

**Figure 4:**
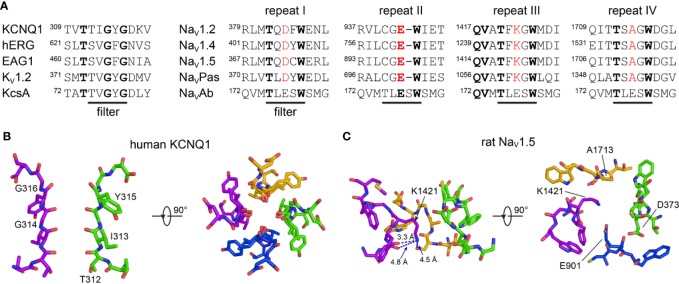
The selectivity filters of K^+^ and Na^+^ channels. **(A)** Multiple sequence alignment of the SF region of selected K^+^ (left) and Na^+^ (right) channels. Conserved amino acids are highlighted in bold. Amino acids belonging to the DEKA signature motif in eukaryotic Na^+^ channels are colored red. **(B)** Side and top view of the SF of human KCNQ1 (PDB: 6UZZ) (Sun and Mackinnon, 2020). Two subunits are omitted for clarity in the left plot. **(C)** Side and top view of the SF of SCN5A (PDB: 6UZ3) (Jiang et al., 2020). Repeat II is omitted for clarity in the left plot.

Once the intracellular gate is opened, K^+^ ions move through the pore along their electrochemical gradient. The backbone carbonyl oxygens of the TIGYG motif in the SF of KCNQ1 (TVGYG in K_V_1.2 and KcsA) (**Figure 4A**) and the sidechain of T312 form four evenly spaced K^+^ binding sites (**Figure 4B**) that facilitate K^+^ movement (Zhou et al., 2001). The arrangement of these oxygens mimics the displaced hydration shell of K^+^, which lowers the transfer energy from the aqueous cavity at the center of the channel to the SF, allowing conduction to occur at rates near the diffusion limit (Morais-Cabral et al., 2001; Zhou et al., 2001).

#### hERG

The cryo-EM structure of hERG shows an open pore and activated VSDs, with the first three gating charges (K1–R3) of S4 located on the extracellular side of the CTC (**Figure 3A**). This is a translocation of one charge fewer than in the activated VSD of KCNQ1 (Sun and Mackinnon, 2017) and Shaker-like K_V_1.2–2.1 (Long et al., 2007) where four gating charges are located above the conserved phenylalanine in the CTC. This observation agrees with gating current measurements suggesting a total charge movement of only ~6 elementary charge units (movement of 1.5 positive charges per S4 helix) for hERG during activation (Zhang et al., 2004), compared to 8 to 9 (2 positive charges per helix) for KCNQ1 (Ruscic et al., 2013) and 12 to 16 (3–4 positive charges per helix) for Shaker-like K_V_ channels (Schoppa et al., 1992; Aggarwal and Mackinnon, 1996; Seoh et al., 1996). However, the ca. 50% lower total gating charge movement for hERG relative to Shaker-like channels is not explained by differences in the number of S4 gating charges, since hERG has a total of five positively-charged residues on S4 and Shaker has six (Pless et al., 2011b). These combined structural and functional data thus imply that S4 translocates less in hERG during activation, resulting in smaller overall VSD conformational changes.

However, the activated VSD of hERG may not be fully defined. While the position of K1–R3 above the CTC in the cryo-EM structure of hERG is consistent with a depolarized VSD, some salt bridge interactions in the VSD have suboptimal geometry, particularly with R4. This is likely due to the limited resolution of the VSD in the final map (approximately 4.5–5.5 Å) (Wang and Mackinnon, 2017), impeding unambiguous determination of sidechain conformations. While cryo-EM has proven to be a powerful structural tool, the resolution is often lower in the periphery of protein structures (Herzik et al., 2019). This can prevent accurate modeling of functional features and lead to discrepancies with experimental data. Molecular dynamics may be a be useful tool in refining cryo-EM structures to mitigate these discrepancies, as is currently being carried out for the hERG structure (Khan et al., 2020).

While a structure of hERG with a closed PD has not yet been determined, we can gain insight into the conformational changes that occur during pore opening using the closed-pore structure of the closely related rat potassium voltage-gated subfamily H member 1 channel (EAG1 or KCNH1) (Whicher and Mackinnon, 2016). In hERG, the intracellular gate is likely constricted by the Q664 side chains in S6, since the radius of the cavity at Q664 is almost 6 Å in the open state hERG structure (Wang and Mackinnon, 2017), while at the corresponding position (Q476) in the EAG1 closed state structure, the pore is at its narrowest (< 1 Å) (Whicher and Mackinnon, 2016). Bending and displacement of the S6 helices is suggested by a glycine residue, located at the same position in both channels, acting as a gating hinge (G648 in hERG, G460 in EAG1).

The SF in hERG is unique among K_v_ channels, containing a GFG motif (**Figure 4A**) in place of the typical GYG motif (Long et al., 2005). The position of the phenylalanine residue in this motif is different from the corresponding tyrosine in other K_v_ channels of known structure (Wang and Mackinnon, 2017). This structural variation may have important implications for fast inactivation in hERG, as discussed below.

#### SCN5A

The mechanism of voltage sensing in Na_V_ channels is thought to be similar to that of K_V_ channels. The S4 helix is the key sensor of transmembrane voltage. Pairing of the positively-charged residues in S4 with polar or negatively-charged residues catalyzes S4 movement from its inward resting-state position to the outward activated state upon membrane depolarization. Recent cryo-EM structures of Na_V_ channels with VSDs in activated (Yan et al., 2017b; Pan et al., 2018; Pan et al., 2019; Shen et al., 2019; Jiang et al., 2020) and resting (Clairfeuille et al., 2019; Wisedchaisri et al., 2019; Xu et al., 2019) conformations uncover a remarkable 10 to 15 Å translation of S4 across the membrane, fully consistent with the “sliding helix” model of VSD activation (Catterall, 1986). A conserved aromatic residue (tyrosine in repeat I, phenylalanine II, III, and IV) on S2 serves as the hydrophobic plug that constricts the S4 gating canal and prevents ion leak through the VSD (Jiang et al., 2020). This hydrophobic plug is mechanistically identical to the corresponding residues in the CTC of K_V_ channels (F167 in KCNQ1, F463 in hERG). However, in contrast to the VSD of KCNQ1 and hERG, the number of basic residues on S4 in SCN5A varies from four (repeat I) to six (repeat IV) (Jiang et al., 2020) (**Figure 3B**). The variation in the number of gating charges and the heterogenous distribution of acidic and polar residues on S1–S3 between KCNQ1, hERG, and SCN5A (**Figure 3B**) may be responsible for their distinct voltage sensitivities and kinetics of VSD activation. In Na_V_ channels, the VSDs of repeats I, II, and III are mainly responsible for channel activation and pore opening, while the VSD of repeat IV is responsible for initiating and maintaining fast inactivation (Chanda and Bezanilla, 2002; Capes et al., 2013; Clairfeuille et al., 2019). This activation process has been studied in detail for the human skeletal muscle channel Na_V_1.4, giving rise to the “asynchronous gating model” (Chanda and Bezanilla, 2002; Capes et al., 2013; Goldschen-Ohm et al., 2013) wherein the S4 segments of repeats I, II, and III move quickly, permitting conductance before activation of VSD_IV_, while S4 movement in repeat IV is slower and represents the rate-limiting step for development of and recovery from inactivation.

The structure and function of the selectivity filter in SCN5A and other Na_V_ channels differs fundamentally from that of K_V_ channels such as KCNQ1 and hERG (**Figure 4**). The SF gate in Na_V_ channels is wider to allow Na^+^ ions to pass in a partially hydrated state (Hille, 1971; Naylor et al., 2016). The extracellular vestibule of Na_V_ channels is lined by negatively-charged residues that recruit Na^+^ ions to the SF. Coordination by both sidechain and backbone carbonyls contribute to the Na^+^ permeation mechanism (Chakrabarti et al., 2013; Ulmschneider et al., 2013; Naylor et al., 2016). Additionally, the SF of eukaryotic Na_V_ channels is formed in a pseudo-symmetric fashion by four short helix-connecting turn motifs from each subunit (**Figure 4A**). Four distinct residues–DEKA, one in each repeat-form the signature motif for Na^+^ selectivity found in all human Na_V_ channel pore-forming repeats (**Figure 4C**). The lysine residue in the DEKA motif (K1419 in SCN5A) confers selectivity for Na^+^ and prevents permeability of Ca^2+^ (Favre et al., 1996). The recent rat SCN5A structure suggests a mechanism by which K1419 contributes to this selectivity of Na^+^ over Ca^2+^, whrein lysine forms a charge delocalization network at a constriction point in the SF. Only Na^+^ ions, which have a compatible size and electric field strength, are able to pass through (Jiang et al., 2020).

### Electromechanical Coupling

#### KCNQ1

In the absence of accessory subunits, KCNQ1 exhibits a constitutive current reflecting close-to-open state transitions even at very negative (~−120 mV) voltages (Ma et al., 2011). Analysis of a large group of KCNQ1 mutants suggests that gating follows an allosteric model (Ma et al., 2011). According to this model, the pore can open independently of the state of the VSD, but VSD activation increases the probability of pore opening. Furthermore, KCNQ1 opening does not require concerted VSD movements (Osteen et al., 2012). Indeed, the VSDs appear to move independently, and pore opening can occur before all VSDs are activated (Osteen et al., 2012), consistent with an allosteric gating model.

**Figure 5:**
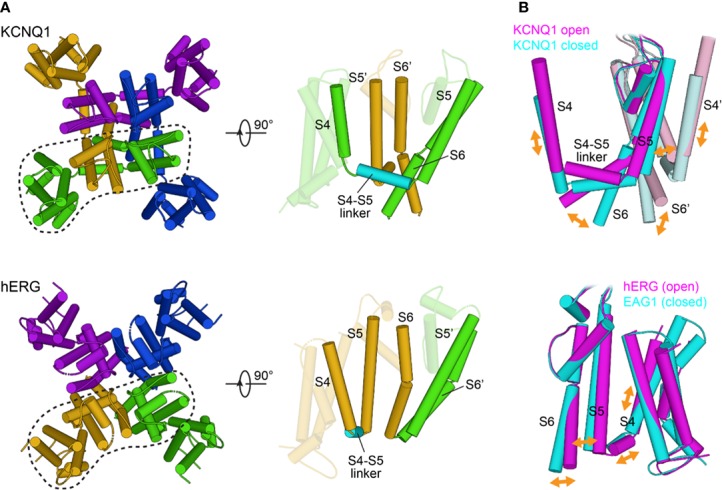
Structural features of electromechanical coupling in the KCNQ1 and hERG channels. **(A)** Left: Extracellular view of KCNQ1 and hERG transmembrane segments S0 to S6. Individual subunits are drawn with different colors. KCNQ1 (PDB: 6UZZ) (Sun and Mackinnon, 2020) has a domain-swapped architecture whereas hERG (PDB: 5VA1) (Wang and Mackinnon, 2017) channels are non-domain-swapped. Right: Cartoon representation of a single subunit and its neighboring pore segments (S5 to S6). In KCNQ1, the VSD and PD are bridged by an extended α-helical S4–S5 linker, whereas in hERG, S4 and S5 are connected by only a short helix. **(B)** Implications for the direction of coupled motions between the VSD and PD based on molecular modeling of KCNQ1 in open and closed conformations (top) and comparison of the open state structure of hERG with the closed state structure of EAG1 (PDB: 5K7L) (Whicher and Mackinnon, 2016) (bottom). In the domain-swapped KCNQ1 channel, the S4 movement is transmitted to the gate through the S4–S5 linker. In hERG and EAG1, the S4 movement is proposed to exert a direct force on the S5–S6 interface to compress or open the channel gate.

Allosteric coupling for KCNQ1 is thought to be mediated by interactions between the VSD and the PD that translate S4 movement to channel opening and closing (**Figure 5**). Studies of KCNQ1 (Boulet et al., 2007; Labro et al., 2011) and of other K_V_ channels (Lu et al., 2001; Lu et al., 2002; Long et al., 2005) have pointed to the interface between the S4–S5 linker and the C-terminal end of S6 (S6_C_) as one important mediator of electromechanical coupling (**Figure 5A**). Certain mutations in the S4–S5 linker (Labro et al., 2011) and S6_C_ (Boulet et al., 2007) slow the opening rate and shift channel activation to more depolarized voltages, while other mutations, specifically at V254 in the S4–S5 linker and at L353 in S6_C_, promote a constitutively open channel. Interestingly, a V254L/L353A double mutant rescued channel closing, suggesting that the S4–S5 linker interacts with S6_C_ to stabilize the closed state. Relocation of the S4–S5 linker during gating abolishes this interaction, releasing tension on S6 and allowing it to kink at the PAG gating hinge in a cantilever-like fashion to promote channel opening (**Figure 5B**). This coupling mechanism is intrinsically weak for KCNQ1, requiring modulation by auxiliary molecules (see below). Recent work has further elucidated the molecular details of S4–S5 linker relocation, revealing a two-stage mechanism involving alternative binding modes of the S4–S5 linker to the PD (Hou et al., 2020). This two-stage mechanism may apply to the majority of domain-swapped K_v_ channels.

#### hERG

The relative positioning of the VSD and PD in hERG (Wang and Mackinnon, 2017) (**Figure 5A**) and the EAG1 channel (Whicher and Mackinnon, 2016) suggests that the mechanism by which movements in the VSD are transduced to pore opening is different from other K_V_ channels (Toombes and Swartz, 2016; Barros et al., 2019). This notion is supported by the finding that cutting the S4–S5 linker in hERG through separate expression of the VSD and PD fails to significantly perturb activation kinetics (Lorinczi et al., 2015). In contrast to the lever mechanism proposed for KCNQ1, lateral S4 movement in hERG toward the pore could both alter S4–S5 linker/S6 interactions and exert force through the S4–S5 linker directly onto S5. Displacement of S5 may be transmitted through the S5–S6 interface for opening or closing of the cytosolic gate (Wang and Mackinnon, 2017) (**Figure 5B**). A structure of hERG in the resting state may provide further insight into the molecular features of this distinct coupling mechanism.

#### SCN5A

Much of our knowledge about the coupling in SCN5A originates from studies of ancestral Na_V_ channels. Comparison of locked resting state structures of the bacterial sodium channel Na_V_Ab (Wisedchaisri et al., 2019) and of the chimeric human Na_V_1.7–VSD_II_–Na_V_Ab channel (Xu et al., 2019) with activated state structures (Yan et al., 2017b; Pan et al., 2018; Pan et al., 2019; Shen et al., 2019; Jiang et al., 2020) provide new insight into the mechanism of electromechanical coupling in Na_V_ channels. The S4–S5 linker appears to undergo movement similar to that of KCNQ1. The linker constrains the S5 and S6 helices in the resting/closed state, with looser interactions in the activated/open state. Coupling between the VSD and PD during pore opening must involve loosening of linker/PD interactions as S4 moves outward as in KCNQ1, albeit with tighter coupling of S4 and S4–S5 linker movement. The direct connection between S4 movement and pore opening may be crucial for rapid activation of sodium channels.

Comparison of the cockroach Na_V_PaS channel structure (Shen et al., 2017), featuring a closed pore and VSDs in distinct activation states, to other eukaryotic Na_V_ channel structures suggests that additional structural shifts may be at work in eukaryotic Na_V_ channels to couple VSD to PD movement. Moreover, the distinct sequence of repeats I to IV (including that of the four S4–S5 linkers) and asynchronous voltage sensor movement in eukaryotic Na_V_ channels (Chanda and Bezanilla, 2002) suggest that distinct interactions couple the VSD of each repeat to the PD. Electromechanical coupling mechanisms may thus be more complex in SCN5A.

### Inactivation

#### KCNQ1

Inactivation in KCNQ1 follows a mechanism distinct from canonical mechanisms (Hou et al., 2017). In the absence of KCNE1, KCNQ1 only partially inactivates, in a manner dependent on the IO and AO open states (Pusch et al., 1998). Differences in VSD-PD coupling between these two states appear to contribute to inactivation. The AO state has a lower coupling efficiency than IO, producing a lower open probability. Transition from IO to AO thus results in partial inactivation because the channel is open but less conductive (Hou et al., 2017). However, association with the KCNE1 accessory β subunit removes KCNQ1 inactivation (Pusch et al., 1998; Seebohm et al., 2003c), making inactivation irrelevant to cardiac KCNQ1 function.

#### hERG

Inactivation plays a critical role in hERG activity in the action potential. Entry into and out of inactivation is both fast and voltage-dependent, properties that maintain the plateau of the action potential (Perry et al., 2015). Additionally, the voltage dependence of inactivation appears to be independent of that of activation (Vandenberg et al., 2006; Cheng and Claydon, 2012), indicating that activation and inactivation may operate through distinct mechanisms. Inactivation in hERG is C-type (Smith et al., 1996), occurring through structural changes in the SF (Herzberg et al., 1998 14243; Hoshi and Armstrong, 2013). While the structural basis of C-type inactivation is not completely understood, work on hERG and other K^+^ channels has provided useful insight. Several residues in the KcsA SF form stabilizing hydrogen bond networks that suppress inactivation (Doyle et al., 1998; Bhate et al., 2010; Cuello et al., 2010; Vandenberg et al., 2012). While these residues are conserved in many K_v_ channels (Whicher and Mackinnon, 2016), they are not present in hERG. This implies that the hERG SF is more liable to collapse, leading to inactivation (Fan et al., 1999; Vandenberg et al., 2012). Indeed, in molecular dynamics simulations, several residues in the hERG SF shift in and out of the pore axis, particularly F627 in the GFG motif (Stansfeld et al., 2008). In the hERG cryo-EM structure, the orientation of F627 is offset compared to other K_v_ channels (Whicher and Mackinnon, 2016), and a hERG S631A mutant, which has an F627 sidechain orientation similar to other structures, does not inactivate (Wang and Mackinnon, 2017). Additionally, mutation of T432 and A443 (corresponding to S620 and S631 in hERG) in non-inactivating EAG1 to serine was sufficient to impart inactivation behavior (Ficker et al., 2001), perhaps due to reorientation of the SF to match that of hERG. These results indicate that the unique positioning of F627 is critical for hERG fast inactivation behavior. C-type inactivation may involve other rearrangements (Loots and Isacoff, 1998), including coupling the SF to motions in S1, S5, and S6 (Ferrer et al., 2011; Wang et al., 2011; Perry et al., 2013a; Perry et al., 2013b).

#### SCN5A

**Figure 6:**
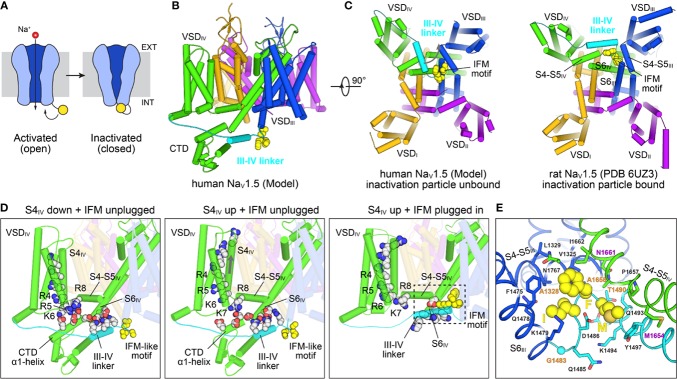
Structural mechanisms of Na_V_ channel inactivation. **(A)** Na_V_ channels transition from an activated open state to a non-conducting inactivated state after depolarization. Inactivation is induced by binding of a C-terminal motif (yellow) to the cytosolic side of the channel leading to pore closure. The cell membrane is indicated with a gray rectangle, with the extracellular (EXT) and intracellular space (INT) labeled. **(B)** Closed state model of SCN5A (Kroncke et al., 2019) highlighting structural elements involved in fast inactivation. The III-IV linker (cyan) connecting S6_III_ (blue) with VSD_IV_ (green) contains the IFM motif (yellow spheres) which is the key structural element responsible for inactivation. The position of the III-IV linker is constrained by the CTD following S6_IV_. **(C)** Comparison of the SCN5A model in **(B)** with a cryo-EM structural model of rat SCN5A (PDB: 6UZ3)(Jiang et al., 2020) in a putative inactivated state viewed from the intracellular side. The III-IV linker undergoes a large shift in the SCN5A structure and the IFM motif is docked into a pocket surrounded by the S4–S5 linkers and S6 helices of repeats III and IV. **(D)** Structural states and transitions proposed to be involved in fast inactivation in Na_V_ channels. Left: Structure of a chimeric Na_V_1.7–Na_V_PaS channel (Clairfeuille et al., 2019) (PDB: 6NT3) with S4_IV_ in a “down” position and an IFM-like motif unbound. Basic sites R5–R8 bridge to conserved acidic residues on the CTD which facilitates binding of the III-IV linker to S6_IV_. Middle: Structure of a chimeric Na_V_1.7–Na_V_PaS channel (Clairfeuille et al., 2019) (PDB: 6NT4) with S4_IV_ in a ‘up’ position and an IFM-like motif unbound. The electrostatic bridge between S4_IV_ and the CTD is broken possibly increasing the positional dynamics of the CTD and III-IV linker. Right: Structure of SCN5A (PDB: 6UZ3) (Jiang et al., 2020) with S4_IV_ in an ‘up’ position and the IFM motif plugged into the cytosolic cavity between repeats III and IV. Note, that the CTD is missing in the cryo-EM structure. **(E)** IFM binding pocket residues on S4–S5_III_, S4–S5_IV_, S6_III_, and S6_IV_ of SCN5A (PDB: 6UZ3) (Jiang et al., 2020). The S6_IV_ helix backbone in the front is not shown for clarity. Residues that are important for fast inactivation and those found in various types of myotonia (Pan et al., 2018) are labeled magenta and orange, respectively.

Fast inactivation in SCN5A halts inward Na^+^ current triggered by cardiac depolarization, permitting subsequent outward currents (e.g. I_Ks_, I_Kr_) to repolarize the cell in preparation for the next action potential (Ghovanloo et al., 2016) (**Figure 6A**). The S4 segment in VSD_IV_ (S4_IV_) is critical for fast inactivation, along with the IFM motif in the III/IV linker (Ahern et al., 2016; Ghovanloo et al., 2016) (**Figures 6B, C**). Roles of individual basic sites of S4_IV_ in Na_v_ inactivation have been elucidated, with mutations in R1 and R2 delaying inactivation onset and mutations in R3 and R4 delaying recovery from inactivation (Nakajima et al., 2019). Furthermore, structures of an engineered human Na_V_1.7–Na_V_PaS channel with VSD_IV_ trapped in a resting state (Clairfeuille et al., 2019) along with the structures of Na_v_PaS (Shen et al., 2017), electric eel (Yan et al., 2017b) and human Na_v_1.4 channels (Pan et al., 2018) have provided new structural insight into the mechanism of fast inactivation in Na_V_ channels (**Figure 6D**). In the resting state R5 on S4_IV_ forms an electrostatic bridge with the α1 helix of the C-terminal cytoplasmic domain (CTD), together with K7 and R8 on the S4–S5 linker. The CTD in turn binds the III/IV linker, sequestering the IFM motif (**Figure 6D**, left plot). VSD_IV_ activation releases the connection between the CTD and the III/IV linker (middle and right plots in **Figure 6D**), permitting the IFM motif to bind to a hydrophobic pocket formed by the S4–S5 linkers and S6 helices of repeats III and IV, along with S5_IV_ (**Figures 6C, E**). The insertion of the IFM motif into this pocket causes a twisting in the S6 helices that closes the gate (Yan et al., 2017b; Pan et al., 2018).

## Regulation of Channel Gating by Intracellular Domains And Auxiliary Molecules

The function of these cardiac channels is tightly regulated to produce currents that faithfully give rise to the cardiac action potential under a host of physiological conditions and prevent early or delayed contractility. We focus here on regulation through the channel cytoplasmic domains as well as regulation by auxiliary (beta) subunits and lipids. Channels are regulated through other mechanisms such as phosphorylation, but are not covered in this review.

### Regulation Involving Cytoplasmic Domains

#### KCNQ1

The C-terminal cytoplasmic domain (CTD) of KCNQ1 contains four alpha helices in lieu of the T1 tetramerization domain common to K_v_ channels outside the KCNQ family (Haitin and Attali, 2008). The proximal HA and HB helices form an antiparallel bundle with an IQ motif in HA and a 1-5-10 motif in HB that together enable calmodulin (CaM) binding (Yus-Najera et al., 2002). When bound, CaM prevents channel inactivation in KCNQ1, but inhibits the opening of other KCNQ family members (KCNQ2–KCNQ5) (Chang et al., 2018). While the N-lobe of CaM may be constitutively bound to both calcium and the proximal HA and HB helices (Ghosh et al., 2006; Bernardo-Seisdedos et al., 2018), the C-lobe of CaM binds calcium only at higher concentrations (Bernardo-Seisdedos et al., 2018). Calcium binding by the C-lobe induces a conformational change in CaM, facilitating KCNQ1 opening (Bernardo-Seisdedos et al., 2018; Chang et al., 2018). The C-lobe interacts with HA and with a loop in the S2–S3 linker (Sun and Mackinnon, 2017). This feature may contribute to the unique mode of CaM modulation of KCNQ1.

The distal helices HC and HD of KCNQ1 form a self-assembling intersubunit coiled-coil motif that promotes channel tetramerization (Yus-Najera et al., 2002; Howard et al., 2007; Sun and Mackinnon, 2017). The distal coiled-coil domain is not only essential for channel tetramerization, but also for subunit specificity, permitting the exclusive formation of KCNQ1 homotetramers (Schwake et al., 2003; Haitin and Attali, 2008; Sachyani et al., 2014). In contrast, other KCNQ family proteins form heteromers (Schwake et al., 2003). Swapping the coiled-coil domain of KCNQ1 with that of KCNQ3 allows the resulting chimera to co-assemble with other KCNQ isoforms and generate channels with altered gating properties (Schwake et al., 2003; Schwake et al., 2006). Residues conferring specific KCNQ1 homotetramer formation have been identified in the HD helix (Schwake et al., 2006; Wiener et al., 2008). Key interactions involve both the hydrophobic core of the assembly and exterior electrostatic interactions, with a number of the residues involved subject to LQTS-associated mutations (Howard et al., 2007; Wiener et al., 2008).

#### hERG

hERG channels contain an N-terminal Per-ARNT-Sim (PAS) domain, which is also present in the N-terminus of many signaling proteins (Henry and Crosson, 2011). An additional C-terminal cyclic nucleotide-binding homology domain (CNBHD) similar to cytoplasmic domains in hyperpolarization-sensitive cyclic nucleotide-gated channels is present, but without ligand-binding properties (Codding and Trudeau, 2019). The PAS and the CNBHD appear to be critical for modulating the kinetics of slow deactivation in hERG (Gustina and Trudeau, 2011). Furthermore, slow deactivation is dependent on the direct interaction of these domains, particularly between R56 of the PAS and D803 of the CNBHD and between N12 of the N-terminal Cap (N-Cap) and E788 of the CNBHD (Ng et al., 2014; Kume et al., 2018). Interactions between the N-Cap and PAS domains with the C-linker also appear to be important for deactivation (Gustina and Trudeau, 2011; Ng et al., 2014), and the extreme N-terminus is likely essential, possibly interacting with the VSD through a patch of positively-charged residues in the N-Cap tail (Muskett et al., 2011). Indeed, the hERG cryo-EM structure corroborates the positioning of the N-Cap tail relative to the VSD (Wang and Mackinnon, 2017). The N-Cap tail may even be constitutively bound to the VSD (Morais Cabral et al., 1998; De La Pena et al., 2011), implying that slow deactivation could involve movement of the PAS toward the plasma membrane to alter this interaction (Barros et al., 2018).

#### SCN5A

Multiple structures of the SCN5A CTD in complex with regulatory factors (Wang et al., 2012; Gabelli et al., 2014; Gardill et al., 2019) have been determined, helping to elucidate how CaM (Johnson et al., 2018) and fibroblast growth factor-homologous factors (FHFs), specifically FGF13, modulate inactivation (Liu et al., 2003; Wang et al., 2012; Musa et al., 2015; Yang et al., 2016). CaM binds the CTD in a calcium-dependent manner (Kim et al., 2004; Hovey et al., 2017; Johnson et al., 2018; Urrutia et al., 2019), altering inactivation kinetics (Yan et al., 2017a; Johnson et al., 2018) and promoting recovery from inactivation (Johnson et al., 2018). In the absence of FHF, the C-lobe of apo-CaM binds to the IQ motif located in an extended helix on the CTD, while the N-lobe binds to the preceding EF hand-like domain (EFL) (Gabelli et al., 2014). With increasing intracellular calcium, holo-CaM has stronger affinity for the SCN5A III/IV linker (Johnson et al., 2018) and the N-lobe disassociates from the EFL (Gardill et al., 2019). These calcium-dependent interactions with the III/IV linker may facilitate CaM modulation of inactivation (Hovey et al., 2017; Johnson et al., 2018). FGF13 slows both inactivation onset and inactivation recovery in SCN5A (Yang et al., 2016), opposing CaM modulation. Importantly, binding of FGF13 to the SCN5A EFL (Wang et al., 2012) prevents binding of the apo-CaM N-lobe to the same domain (Wang et al., 2012). The EFL itself also adopts different orientations when bound to FGF13 (Wang et al., 2012) or CaM (Gabelli et al., 2014). Because the EFL also binds to the III/IV linker containing the IFM inactivation motif (Shen et al., 2017; Clairfeuille et al., 2019), EFL conformational changes upon binding of CaM or FGF13 may contribute to inactivation modulation by these proteins.

SCN5A also contains two cytoplasmic loops linking repeats I and II and repeats II and III, respectively. Unlike the III/IV linker, these loops are around 200 residues long (Pan et al., 2018) and likely disordered, based on the absence of these loops in cryo-EM structures. However, important regulatory events such as phosphorylation (Marionneau et al., 2012; Iqbal et al., 2018) and cofactor binding (Wu et al., 2008) that modulate channel properties have been identified in these loops.

### Auxiliary Beta Subunits

#### KCNQ1

KCNQ1 co-assembles with a family of single span membrane proteins (KCNE1–5) (Abbott, 2014), and this interaction appears to be tissue–specific. In the heart, KCNQ1 is complexed with KCNE1, and the KCNQ1-KCNE1 channel exhibits greater single-channel conductance, opening at more positive potentials, and delayed activation compared to KCNQ1 alone (Barhanin et al., 1996; Sanguinetti et al., 1996). This heteromultimeric channel also exhibits altered Rb^+^/K^+^ selectivity (Pusch et al., 2000) and loss of inactivation (Pusch et al., 1998; Pusch et al., 2000). These properties are essential for the generation of the slow delayed rectifier current (I_Ks_) in repolarizing cardiomyocytes. In gastric parietal cells, KCNQ1 co-assembles with KCNE2 to create constitutive K^+^ currents essential for gastric acid secretion (Heitzmann et al., 2004; Roepke et al., 2006). In intestinal epithelial cells KCNQ1 complexes with KCNE3 to allow K^+^ recycling for trans-epithelial chloride ion secretion (Preston et al., 2010). In contrast to KCNE1, KCNE2 and KCNE3 render KCNQ1 constitutively open (Schroeder et al., 2000; Tinel et al., 2000).

**Figure 7:**
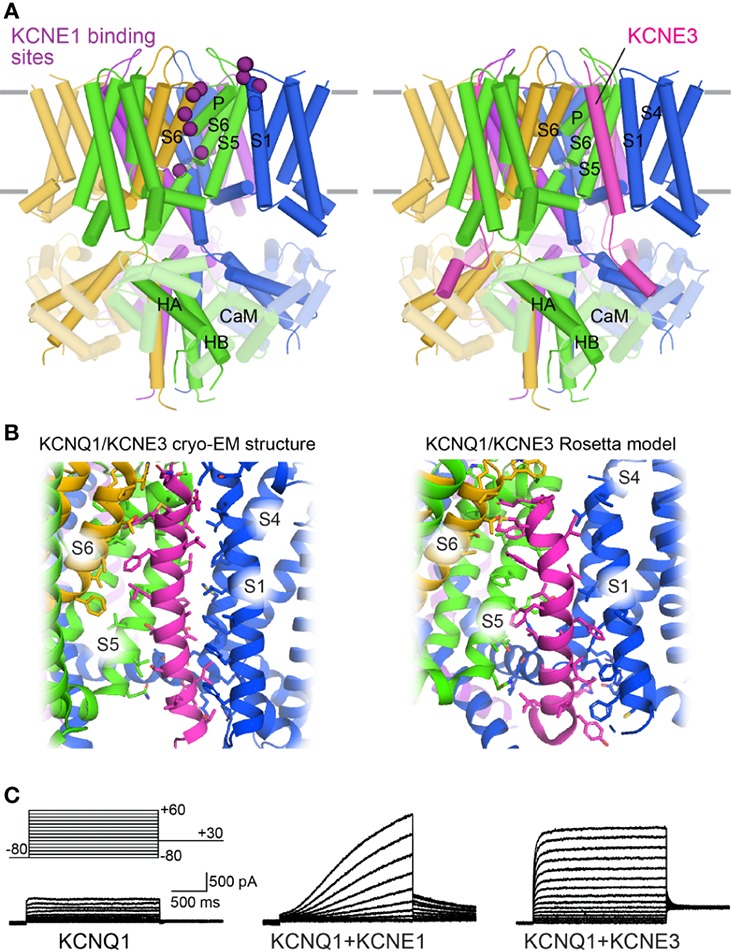
Binding sites of KCNE proteins on the KCNQ1 channel. **(A)** Left: Mapping of putative KCNE binding sites onto the human KCNQ1 structure (PDB: 6V00) (Sun and Mackinnon, 2020). The position of residues identified to interact with KCNE1 (purple) are indicated by spheres. Right: Cryo-EM structure of human KCNQ1 with CaM and KCNE3 (PDB: 6v00)(Sun and Mackinnon, 2020). **(B)** KCNQ1/KCNE3 interaction sites. Left: 6V00. Right: KCNQ1/KCNE3 Rosetta model (Kroncke et al., 2016). Interactions sites were calculated with the InterResidues Python script in PYMOL (The PyMOL Molecular Graphics System, Version 2.2 Schrödinger, LLC.). Interaction positions indicated with sticks. **(C)** Example current traces of KCNQ1 alone or with KCNE proteins. Adapted with permission from (Van Horn et al., 2011).

Due to the critical role of the KCNQ1-KCNE1 channel in the cardiac action potential, many research groups have probed the structural interaction between these proteins (Tapper and George, 2001; Xu et al., 2008; Chung et al., 2009; Strutz-Seebohm et al., 2011; Wang et al., 2011; Chan et al., 2012; Li et al., 2014). Combined results indicate that the transmembrane helix of KCNE1 binds in the cleft between neighboring KCNQ1 subunits and interacts with both the VSD and PD. These binding sites overlap the binding cleft of KCNE3 (Sun and Mackinnon, 2020), indicating that KCNE1 may bind to the same site (**Figure 7A**). In the human KCNQ1-KCNE3 cryo-EM structure (Sun and Mackinnon, 2020) and an earlier Rosetta model of the complex (Kroncke et al., 2016), this cleft is formed by three KCNQ1 subunits (**Figure 7B**). KCNE3 contacts the cytoplasmic half of S5 in one subunit, the extracellular side of S6 in a second one, and along the entire length of S1 and the cytoplasmic side of S4 in a third subunit. Importantly, KCNE1 and KCNE3 produce KCNQ1 currents with different voltage dependencies and gating kinetics (**Figure 7C**). Given the possibility of a common binding cleft, differential modulation of KCNQ1 by KCNE1 and KCNE3 may involve different sidechain interactions within this cleft.

Mutations in the KCNE1 transmembrane helix, particularly at F57, T58, and L59 (Melman et al., 2001; Melman et al., 2002), alter the ability of this subunit to modulate the function of KCNQ1. The effect of this ‘activation’ triplet on KCNQ1 gating is altered by mutations at residues S338, F339, F340, and A341 in S6 (Melman et al., 2004; Panaghie et al., 2006; Strutz-Seebohm et al., 2011; Li et al., 2014), suggesting a functional link between these residues. However, modeling studies fail to indicate direct contact between the activation triplet on KCNE1 and these S6 sites (Kang et al., 2008; Gofman et al., 2012; Xu et al., 2013), suggesting that the functional coupling may be mediated allosterically. Interestingly, KCNE1 also affects S4 movement (Rocheleau and Kobertz, 2008; Wu et al., 2010a; Nakajo and Kubo, 2014) and shifts VSD voltage-dependence of activation to more negative voltages (Osteen et al., 2010; Ruscic et al., 2013; Barro-Soria et al., 2014). A model of KCNE1 regulation has recently been proposed, in which KCNE1 alters VSD-PD coupling interactions to suppress the IO state and modulate the AO state (Zaydman et al., 2014). This model accounts for many of the observed effects of KCNE1 on KCNQ1, including KCNE1-induced inhibition of inactivation. However, this model is not structurally elaborated.

#### hERG

Previous studies indicate that KCNE1 (Mcdonald et al., 1997) and KCNE2 (Abbott et al., 1999) can interact with and modulate hERG function. However, whether this interaction occurs under physiological conditions is debated (Weerapura et al., 2002; Anantharam and Abbott, 2005; Abbott et al., 2007). Although KCNE1 and KCNE2 alter hERG gating kinetics *in vivo* (Mcdonald et al., 1997; Mazhari et al., 2001), co-expression of KCNE2 with hERG *in vitro* does not reproduce the native I_Kr_ current (Weerapura et al., 2002). This suggests that KCNE1 and KCNE2 may not be essential for hERG channel function or that additional factors are required for KCNE-hERG interaction in cardiac cells. The latter possibility is supported by the observation that mutations in KCNE2 may predispose patients to drug-induced LQTS (Abbott et al., 1999; Sesti et al., 2000). As hERG is particularly drug-sensitive, this finding suggests that hERG may, in fact, be modulated by KCNEs in native tissue. Additionally, a T10M mutation in KCNE2 causes arrhythmia induced by auditory stimulation, a known trigger of LQT2, (Gordon et al., 2008). Further studies are needed to resolve these conflicting results and clarify the role of KCNE proteins in regulation of hERG function.

#### SCN5A

In humans there are five Na_V_-β-subunit protein isoforms encoded by four genes, SCN1B-SCN4B (β1 to β4) (Detta et al., 2015; O’malley and Isom, 2015). All are expressed in the heart and have been shown to associate with SCN5A in heterologous conditions (Makita et al., 1996; Dhar Malhotra et al., 2001; Malhotra et al., 2004; Medeiros-Domingo et al., 2007; Watanabe et al., 2009; Valdivia et al., 2010). β1 to β4 are single-span transmembrane proteins containing an extracellular N-terminal immunoglobulin (Ig) domain, while β1B, a splice variant of SCN1B, lacks the transmembrane domain (Brackenbury and Isom, 2011). Generally, β-subunits modulate the biophysical properties and cell surface expression of Na_V_ channels in heterologous cells (Calhoun and Isom, 2014). β1 and β3 interact non-covalently with Na_V_ channels (Meadows et al., 2001), while β2 and β4 are covalently bound through cysteine bonds between the extracellular Ig domain and channel pore loops. However, modulation of SCN5A by β-subunits has been difficult to assess. Varying effects of β1 (Qu et al., 1995; Dhar Malhotra et al., 2001; Baroni et al., 2014; Zhu et al., 2017) as well as β3 (Hu et al., 2009; Valdivia et al., 2010; Wang et al., 2010) on SCN5A have been reported. The rat SCN5A structure provides a potential explanation for this difficulty, as SCN5A is missing a cysteine residue critical for covalent interaction with β2, and contains a glycosylation site that may sterically occlude β1 interaction (Jiang et al., 2020). SCN5A may not bind tightly with any β-subunit. Nevertheless, the potential importance of β-subunits in SCN5A modulation has been suggested by arrhythmia-associated mutations in all four β-subunit genes, including mutations causing Brugada syndrome (Watanabe et al., 2008; Hu et al., 2009; Hu et al., 2012), LQT3 (Medeiros-Domingo et al., 2007; Riuro et al., 2014), and atrial fibrillation (Watanabe et al., 2009; Olesen et al., 2011). However, a recent review of genetic evidence supporting these associations has disputed the clinical validity of β subunits as monogenic causes of arrhythmia syndromes (Hosseini et al., 2018; Adler et al., 2020).

SCN5A subcellular localization also contributes importantly to channel regulation. SCN5A channels in cardiac tissue are localized at the lateral membranes and at the anchoring junction between cardiomyocytes (the intercalated disc). These expression patterns give rise to distinct sets of protein-protein interactions and biophysical properties (Lin et al., 2011; Shy et al., 2013). At the lateral membrane SCN5A interacts with the dystrophin/syntrophin multicomplex, but at the intercalated disc SCN5A interacts with ankyrin-G (Lemaillet et al., 2003; Mohler et al., 2004), which links the channel to cytoskeletal proteins such as actin and the desmosomal protein plakophilin-2 (Makara et al., 2014). Intriguingly, the functional properties of SCN5A channels between these two pools also differ: SCN5A at the lateral membrane has smaller current amplitude, distinct voltage-dependence, and slower recovery from inactivation as compared to channels in the intercalated disc (Lin et al., 2011). The functional implications of these differences are not yet clear and remain an active area of study.

### Lipid Molecules

#### KCNQ1

**Figure 8:**
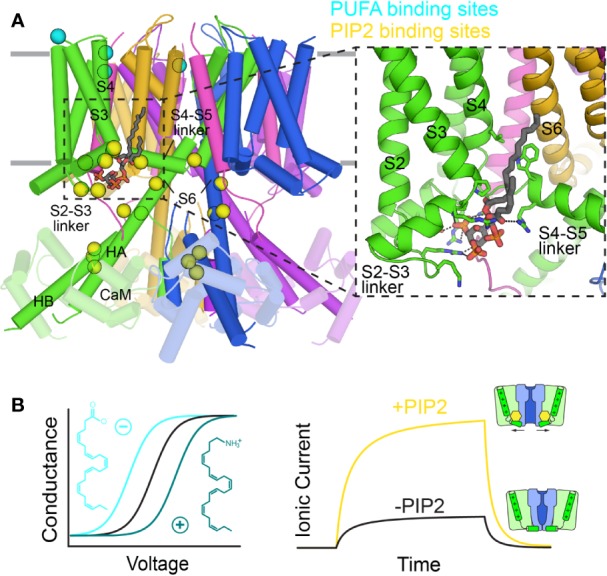
Binding sites of lipids on the KCNQ1 channel. **(A)** Mapping of positions that have been implicated in the regulation of KCNQ1 by PUFAs (cyan) and PIP_2_ (yellow) onto the PIP_2_-bound KCNQ1 structure (6V01) (Sun and Mackinnon, 2020). Inset: the PIP_2_ cryo-EM binding site. **(B)** Left: Effect of the PUFA head group charge on the voltage dependence of KCNQ1 conductance (inspired from results in Liin et al., 2015). Right: PIP_2_ depletion reduces KCNQ1 ionic currents due to decreased VSD-PD coupling.

KCNQ1 and other KCNQ channels are dependent on phosphatidyl-4,5-bisphosphate (PIP_2_) for function (Loussouarn et al., 2003; Zaydman et al., 2013; Taylor and Sanders, 2017), and are inhibited upon stimulation of G_q_- and G_11_-protein coupled receptors, which trigger phospholipase C-catalyzed PIP_2_ hydrolysis (Selyanko et al., 2000; Loussouarn et al., 2003; Zhang et al., 2003) (**Figure 8B**). Growing evidence suggests that PIP_2_ acts as a coupling element for KCNQ1, enhancing weak allosteric interactions between the VSD and PD (Vardanyan and Pongs, 2012; Zaydman et al., 2013; Kasimova et al., 2015; Cui, 2016). Structure-function studies, confirmed by the human KCNQ1/PIP_2_ structure, localize PIP_2_ binding to the cleft between neighboring channel subunits, with interactions involving mostly positively-charged residues in the S2–S3 linker, S4–S5 linker, and S6_C_ (**Figure 8A**) (Thomas et al., 2011; Zaydman et al., 2013; Eckey et al., 2014; Chen et al., 2015; Sun and Mackinnon, 2020). This binding site seems well-suited to modulate coupling of VSD movement to the activation gate. Questions remain regarding the mechanism of PIP_2_ regulation of VSD-PD coupling, PIP_2_:KCNQ1 stoichiometry, and binding site differences between the activated and resting states.

PIP_2_ also binds to the CTD at a site shared with CaM (Tobelaim et al., 2017b). These two regulators may competitively regulate KCNQ1 at this site (Tobelaim et al., 2017a). Indeed, in the human KCNQ1 cryo-EM structure, CaM exhibits a nearly 180° rotation when PIP_2_ is bound, losing contact with the S2–S3 linker (Sun and Mackinnon, 2020). Additionally, S6 and HA in KCNQ1 form a single helix in the open channel. These structural shifts point to an interplay between PIP_2_ and CaM, but does not clarify the nature of this interaction, as no PIP_2_ density was seen at the CTD binding site in the cryo-EM structure. The details of the coordination between CaM and PIP_2_, and its role in channel function, remain to be elucidated.

Polyunsaturated fatty acids (PUFAs) also modulate KCNQ1 function (Taylor and Sanders, 2017). While PUFAs generally inhibit ion channel current (Boland and Drzewiecki, 2008), the KCNQ1-KCNE1 channel is a notable exception. The I_Ks_ current is enhanced by docosahexaenoic acid (DHA) and, to a lesser extent, oleic acid (Doolan et al., 2002) by shifting the conductance-vs-voltage curve (GV) to more negative voltages. Interestingly, the charge of the head group determines the direction of the (GV) shift (**Figure 8B**): negatively-charged DHA causes a negative shift, a neutral head group has no effect, and a positively-charged one shifts the (GV) curve to positive potentials, reducing channel function (Liin et al., 2015). Negative head group charge and a polyunsaturated acyl chain appear to be required for channel activation. The PUFA binding site in KCNQ1 appears to involve residues in the extracellular S3–S4 loop, R1 and R2 on S4, and the PD (**Figure 8A**), as mutations in these regions either reduce the PUFA effect or completely abolish the (GV) shift (Liin et al., 2015; Liin et al., 2018).

#### hERG

As with KCNQ1, the function of hERG is upregulated by PIP_2_ by means of increased current amplitude, a hyperpolarizing shift in voltage-dependence of activation, as well as faster activation and slower inactivation rates (Bian et al., 2001). Stimulation of G_αq_-protein coupled receptors also suppresses the I_Kr_ current through PIP_2_ depletion (Bian et al., 2001; Bian et al., 2004). Binding of PIP_2_ to hERG likely localizes to a cluster of basic residues (R883–Q900) C-terminal to the CNBHD, as substitution of these residues to neutral or negatively-charged amino acids prevented PIP_2_ effects on hERG function and abolished PIP_2_ binding (Bian et al., 2004). Unfortunately, these residues are not resolved in the available hERG structure (Wang and Mackinnon, 2017).

#### SCN5A

In contrast to KCNQ1, PUFAs such as eicosapentaenoic acid (EPA), docosahexaenoic acid (DHA), linoleic acid (LA), and α-linolenic acid (ALA) suppress I_Na_ current in a concentration-dependent manner by shifting the voltage dependence of I_Na_ inactivation to more hyperpolarized potentials (Kang et al., 1995; Kang et al., 1997; Leifert et al., 1999). EPA also accelerates the transition from the resting state to the inactivated state and slows recovery from inactivation (Xiao et al., 1998; Isbilen et al., 2006). However, the effect was reduced by β1 subunit expression (Xiao et al, 2000). Furthermore, N406K renders SCN5A less sensitive to inhibition by EPA, an effect strengthened by β1 subunit expression (Xiao et al., 2001). The structural basis for PUFA binding and modulation is not well understood.

## Channel Dysfunction in Congenital Long QT Syndrome

Alterations in the action potential disturb impulse propagation and cause reentry (Kleber Ag, 2004), whereby the impulse re-stimulates the heart tissue that generated it. Reentry promotes cardiac arrhythmias and predisposes to sudden unexplained death (Skinner et al., 2019). LQTS is a prevalent cause of such events, with an estimated population prevalence of approximately 1:2500 (Schwartz et al., 2009).

LQTS is characterized by a prolonged rate-corrected QT interval in patient electrocardiograms (ECGs), indicative of impaired repolarization (Schwartz et al., 2012). In a large proportion of cases, QT interval prolongation is the result of either a loss-of-function in KCNQ1 or hERG, or a gain-of-function in the SCN5A channel (Moss and Kass, 2005; Schwartz et al., 2012; Earle et al., 2013; Skinner et al., 2019). Mutations in these genes confer distinct subtypes of LQTS (denoted LQT1–3, respectively), each with unique ECG features, risk factors, arrhythmia triggers, and responsiveness to β-adrenergic receptor blockers, the most common LQTS treatment (Ackerman, 2005; Moss and Kass, 2005; Skinner et al., 2019; Wallace et al., 2019). Below, the function of each ion channel in the cardiac action potential is discussed, along with how each can cause action potential dysfunction in LQTS.

### Channel Function in the Cardiac Action Potential and Dysfunction in LQTS

#### KCNQ1

In complex with KCNE1, KCNQ1 conducts I_Ks_, which helps shape the plateau and repolarization phases of the action potential, in part by counteracting calcium influx (Skinner et al., 2019). Due to slow activation, the KCNQ1-KCNE1 channel is only slightly open during the plateau phase, with I_Ks_ increasing slowly in a nearly linear fashion (**Figure 7C**) until the repolarization phase is reached and the channel becomes fully activated (**Figures 1B, C**) (Moss and Kass, 2005). KCNQ1-KCNE1 channels do not inactivate (Pusch et al., 1998), conducting current throughout activation and deactivation. The channel slowly deactivates, shaping the tail of the repolarization phase and mediating return to the resting potential (Moss and Kass, 2005; George, 2013; Skinner et al., 2019). Thus, loss-of-function in KCNQ1 prolongs repolarization primarily by preventing completion of the repolarization phase, broadening the tail of the T-wave (**Figure 1A**) and causing type 1 LQTS (LQT1). (Ackerman, 2005; Tester and Ackerman, 2014; Skinner et al., 2019; Wallace et al., 2019). The effects of a KCNQ1 mutation are exacerbated upon sympathetic activation of β-adrenergic receptors due to I_Ks_ potentiation, while resting heart rates show few perturbations (Shimizu and Antzelevitch, 1998). The QT interval is unable to shorten in response to this stimulation, making physical exertion accompanied by heightened sympathetic nervous system activity a prevalent LQT1 trigger (Schwartz et al., 2012; Bohnen et al., 2017).

#### hERG

hERG makes up the other primary inward rectifier current I_Kr_, which acts in both the plateau and the repolarization phase (**Figures 1B, C**) (Moss and Kass, 2005; Skinner et al., 2019). As the channel activates, rapid entry into and out of inactivation (due to SF fluctuations) creates a persistent current through the plateau phase until slow deactivation, regulated by the PAS and CNBHD domains, closes the channel during the repolarization phase. While both hERG and KCNQ1 are conductive during the plateau and repolarization phases, hERG has a higher unitary conductance than KCNQ1 in both phases (Moss and Kass, 2005; George, 2013). The resulting I_Kr_ current thus supplies a greater proportion of the overall I_K_ current (Cheng and Kodama, 2004), with loss-of-function in hERG (LQT2) correlating with a lower T-wave amplitude in contrast to the normal T-wave amplitude associated with LQT1. Additionally, LQT2 is often triggered at rest (Skinner et al., 2019), consistent with the greater hERG contribution to I_K_. However, while I_Kr_ is usually more prominent (Cheng and Kodama, 2004), the relative density of I_Kr_ and I_Ks_ vary by ventricular cell type (Liu and Antzelevitch, 1995; Viswanathan et al., 1999).

#### SCN5A

SCN5A produces the I_Na_ current that shapes the initial upstroke of the cardiac action potential (Skinner et al., 2019), initiating depolarization. SCN5A is rapidly activated through asynchronous motion of the VSD’s in repeats I-III coupled to pore opening, then quickly inactivates through interaction of the IFM motif with the channel, resulting in a brief inward spike of sodium current that depolarizes the cell (Ghovanloo et al., 2016) (**Figures 1B, C**). Unlike KCNQ1 and hERG, LQT3 is conferred by SCN5A gain-of-function mutations that cause a “leaky” inward sodium current (Wilde and Amin, 2018; Skinner et al., 2019). The functional I_Kr_ and I_Ks_ outward currents are unable to compensate for the persistent inward I_Na_ current, prolonging entry into and completion of the repolarization phase and giving rise to LQT3. As in LQT2, LQT3-related arrhythmias occur most often at rest, rather than in response to stress or exercise (Schwartz et al., 2012). It should be noted that while loss-of-function mutations for SCN5A are not relevant to LQTS, they are prevalent in other channelopathies, particularly Brugada syndrome (Wilde and Amin, 2018; Skinner et al., 2019).

### Molecular Mechanisms of LQTS

**Figure 9:**
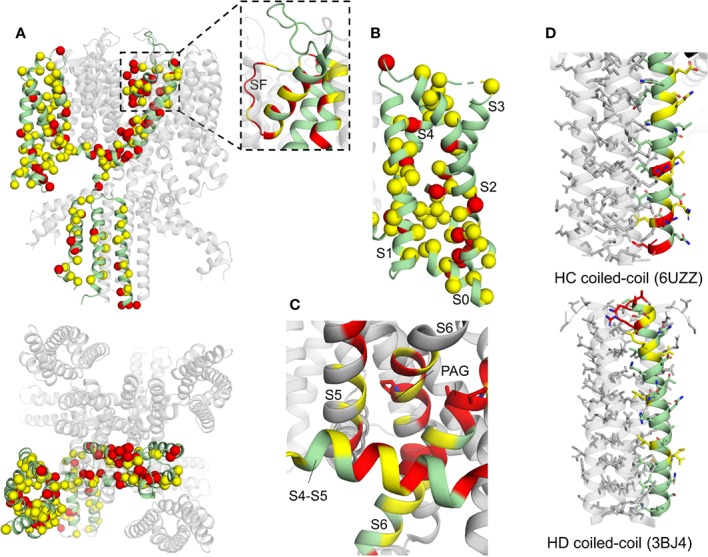
Human KCNQ1 structure with deleterious mutation sites indicated. **(A)** Full KCNQ1 structure (PDB: 6UZZ) (Sun and Mackinnon, 2020). Yellow sites denote locations of a single deleterious mutation, while red indicates sites with multiple mutations identified. Spheres indicate C-alpha positions of mutation sites. Subunit with LQT1 mutations sites mapped is colored green. Inset: of SF and pore helix. **(B)** KCNQ1 VSD. **(C)** S4–S5 linker (S4–S5) interaction site close-up. One subunit is colored green and the others in gray, for clarity. The PAG motif (PAG) is indicated with sticks. **(D)** Top: HC coiled-coil domain. Bottom: HD coiled-coil domain (PDB: 3BJ4) (Wiener et al., 2008). Residue side chains are indicated with sticks.

**Figure 10:**
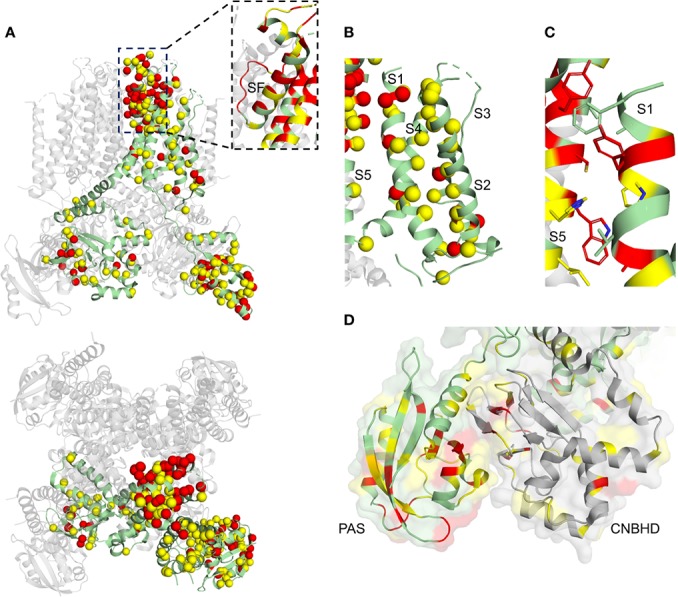
hERG structure with deleterious mutation sites indicated. **(A)** Full hERG structure (PDB: 5VA1) (Wang and Mackinnon, 2017). Yellow sites denote locations of a single deleterious mutation, while red indicates sites with multiple mutations identified. Spheres indicate C-alpha positions of mutation sites. Subunit with LQT2 mutations sites mapped is colored green. Inset: close-up of SF and pore helix. **(B)** hERG VSD. **(C)**. Close-up of the S1/S5 interface. Residues at the interface are indicated with sticks **(D)** N-terminal PAS (dark gray) and C-terminal CNBHD (light gray) domain interface, cartoon representation with surface representation overlay. One subunit is green and the other gray, for clarity.

**Figure 11:**
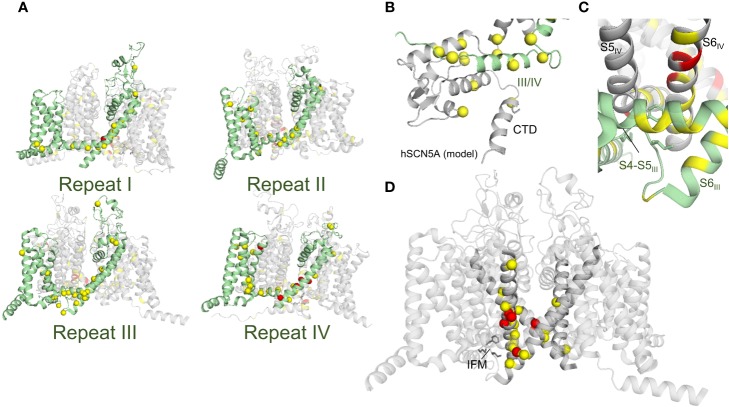
Rat SCN5A structure with deleterious mutation sites indicated. **(A)** Full SCN5A structure (PDB: 6UZ3) (Jiang et al., 2020). Yellow sites denote locations of a single deleterious mutation, while red indicates sites with multiple mutations identified. Spheres indicate C-alpha positions of mutation sites. Side views of individual repeats are shown with 90° rotations between panels. In each panel, one repeat is colored green and LQT3 mutation sites for that repeat are mapped. **(B)** C-terminal domain with bound III/IV linker (human SCN5A model: [(Kroncke Bm, 2019)]. **(C)** Putative inactivation gate. IFM motif is indicated with sticks. **(D)** SCN5A constriction site. S6 helices are shown at full opacity, with the IFM motif indicated with sticks.

Mutations in KCNQ1, hERG, and SCN5A can lead to LQTS by perturbing channel function through distinct molecular mechanisms. Structural studies of these channels can help tease apart these molecular mechanisms for a more detailed understanding of LQTS. We have mapped LQTS mutations identified as pathogenic according to ClinVar (Harrison et al., 2016) and HGMD (Stenson et al., 2012) databases onto the human KCNQ1 (Sun and Mackinnon, 2020) and hERG structures (Wang and Mackinnon, 2017), as well as a recent model of SCN5A (Kroncke et al., 2019) and the rat SCN5A homolog (Jiang et al., 2020) (**Figures 9**–**11**). The list of curated mutations is found in **Table 1**: 261 mutations in KCNQ1, 320 mutations in hERG, and 122 mutations in SCN5A. Examination of these mutation sites provides some insight into the channel mechanisms that may be commonly disrupted in LQT1–3.

**Table 1:** LQTS mutations in KCNQ1, hERG, and SCN5A.

KCNQ1				hERG				SCN5A			
Mutant	Database	Mutant	Database	Mutant	Database	Mutant	Database	Mutant	Database	Mutant	Database
A58P*	HGMD	T311I	HGMD	G6R	HGMD	G572R	ClinVar, HGMD	G9V*	HGMD	G1329S	HGMD
S66F*	HGMD	T312S	HGMD	G6V	HGMD	G572S	HGMD	R18W*	HGMD	A1330P	HGMD
T96R*	HGMD	T312I	ClinVar, HGMD	T13N	HGMD	G572V	HGMD	A29V*	HGMD	A1330T	HGMD
T104I	HGMD	I313M	HGMD	D16A	HGMD	M574V	HGMD	E30G*	HGMD	P1332L	HGMD
Q107H	HGMD	G314A	HGMD	R20G	HGMD	E575G	HGMD	P52S*	HGMD	S1333Y	HGMD
R109L	HGMD	G314D	HGMD	F22S	HGMD	E575K	HGMD	R53Q*	HGMD	L1338V	HGMD
Y111C	ClinVar, HGMD	G314C	HGMD	F22Y	HGMD	R582C*	ClinVar	R104G*	HGMD	A1357V	HGMD
L114P	HGMD	G314R	ClinVar, HGMD	S26I	HGMD	G584S	HGMD	A110T*	HGMD	G1391R	HGMD
E115G	HGMD	G314S	ClinVar, HGMD	R27P	HGMD	G584V	HGMD	V113I*	HGMD	A1428S	HGMD
P117L	ClinVar, HGMD	Y315N	HGMD	K28E	HGMD	W585C	HGMD	S115G*	HGMD	S1458Y	ClinVar, HGMD
C122Y	ClinVar, HGMD	Y315C	HGMD	F29L	HGMD	L586M	HGMD	I176M	HGMD	N1472S	HGMD
Y125D	HGMD	Y315H	HGMD	F29S	HGMD	N588D	HGMD	A185T	HGMD	F1473C	HGMD
F127L	HGMD	Y315F	HGMD	I31S	HGMD	G590V	HGMD	I239V	HGMD	F1473S	HGMD
L131P	HGMD	Y315S	HGMD	I31T	ClinVar, HGMD	I593T	ClinVar	V240M	HGMD	Q1476R	HGMD
I132L	HGMD	G316E	HGMD	A32T	HGMD	I593K	ClinVar, HGMD	Q245K	HGMD	G1481E	HGMD
V133I	HGMD	G316V	HGMD	N33T	HGMD	I593R	ClinVar, HGMD	V258A	HGMD	T1488R	HGMD
L134P	ClinVar, HGMD	G316R	ClinVar, HGMD	R35W	HGMD	I593V	HGMD	R340Q	HGMD	Y1495S	HGMD
C136F	HGMD	D317N	ClinVar, HGMD	V41F	HGMD	G594D	HGMD	A385T	HGMD	K1505N	HGMD
L137F	ClinVar, HGMD	D317G	HGMD	V41A	HGMD	K595N	HGMD	I397T	HGMD	T1544P	HGMD
S140R	HGMD	D317Y	HGMD	I42N	HGMD	K595E	HGMD	L404Q	HGMD	L1560F	HGMD
E146G	HGMD	K318N	ClinVar, HGMD	Y43C	HGMD	P596H	ClinVar, HGMD	N406K	HGMD	I1593M	HGMD
E146K	HGMD	P320A	HGMD	Y43D	HGMD	P596A	ClinVar, HGMD	L409P	HGMD	F1594S	HGMD
A150G	HGMD	T322K	HGMD	C44F	HGMD	P596R	HGMD	L409V	HGMD	V1597M	HGMD
T153M	HGMD	T322A	ClinVar, HGMD	C44W	HGMD	P596L	HGMD	V411M	ClinVar, HGMD	S1609W	HGMD
F157C	HGMD	T322M	ClinVar, HGMD	C44Y	HGMD	P596S	HGMD	A413E	HGMD	R1623Q	ClinVar, HGMD
E160K	HGMD	G325R	ClinVar, HGMD	N45D	HGMD	P596T	HGMD	R504T*	HGMD	R1623L	HGMD
E160V	HGMD	G325E	HGMD	N45S	HGMD	Y597C	HGMD	M506K*	HGMD	R1626P	HGMD
G168R	ClinVar, HGMD	G325W	HGMD	N45Y	HGMD	G604D	HGMD	F530V*	HGMD	R1644H	ClinVar, HGMD
T169R	HGMD	S338F	HGMD	D46Y	HGMD	G604S	ClinVar, HGMD	D536H*	HGMD	L1646R	HGMD
T169K	HGMD	F339S	HGMD	G47D	HGMD	P605S	HGMD	R569W*	HGMD	L1650F	HGMD
E170G	HGMD	F339V	HGMD	G47V	HGMD	S606F	HGMD	Q573E*	HGMD	M1652R	HGMD
V173D	HGMD	F340L	HGMD	C49Y	HGMD	S606P	HGMD	G579R*	HGMD	M1652T	HGMD
R174C	ClinVar, HGMD	A341G	HGMD	G53R	HGMD	D609G	HGMD	P637L*	HGMD	P1725L	HGMD
R174H	ClinVar, HGMD	A341V	ClinVar, HGMD	G53D	HGMD	D609H	HGMD	E654K*	HGMD	A1746T	HGMD
W176R	HGMD	A341E	ClinVar, HGMD	G53S	HGMD	D609N	ClinVar, HGMD	A665S*	HGMD	I1758V	HGMD
A178T	HGMD	L342F	HGMD	G53V	HGMD	D609Y	HGMD	R689C*	HGMD	L1761H	HGMD
A178P	ClinVar, HGMD	P343R	HGMD	Y54H	HGMD	K610N	HGMD	G709V*	HGMD	L1761F	HGMD
K183R	HGMD	P343L	HGMD	S55L	ClinVar, HGMD	Y611D	HGMD	T731I	HGMD	V1763L	HGMD
K183M	HGMD	P343S	HGMD	R56Q	HGMD	Y611H	ClinVar, HGMD	Q750R	HGMD	V1763M	ClinVar, HGMD
Y184C	HGMD	A344E	HGMD	A57P	HGMD	V612L	HGMD	Q779K	HGMD	M1766L	ClinVar, HGMD
Y184H	HGMD	A344V	ClinVar, HGMD	E58A	HGMD	V612M	HGMD	R800L*	HGMD	M1766V	HGMD
Y184S	HGMD	G345R	ClinVar, HGMD	E58D	HGMD	T613A	HGMD	R808P	HGMD	Y1767C	HGMD
G186R	HGMD	G345E	ClinVar, HGMD	E58G	ClinVar, HGMD	T613K	HGMD	F816Y	HGMD	I1768V	ClinVar, HGMD
G189A	HGMD	G345A	HGMD	E58K	HGMD	T613M	ClinVar, HGMD	L828V	HGMD	L1772V	HGMD
G189E	HGMD	G345V	HGMD	Q61R	HGMD	A614V	ClinVar, HGMD	N834D	HGMD	N1774D	HGMD
G189R	ClinVar, HGMD	L347P	HGMD	R62Q	HGMD	L615F	HGMD	G840R	HGMD	E1781G	HGMD
R190Q	ClinVar, HGMD	S349W	ClinVar, HGMD	C64W	HGMD	Y616C	HGMD	T843A	ClinVar, HGMD	E1784K	HGMD
R190L	HGMD	S349P	HGMD	C64Y	HGMD	F617L	HGMD	Q912R	HGMD	D1790G	HGMD
R190W	HGMD	G350R	HGMD	T65P	ClinVar, HGMD	F617V	HGMD	S941N	ClinVar	Y1795C	ClinVar, HGMD
L191P	HGMD	G350V	HGMD	C66G	HGMD	T618S	HGMD	Q960K*	HGMD	P1824A	HGMD
R192P	HGMD	F351L	ClinVar	F68L	HGMD	S620N	HGMD	R975W*	HGMD	D1839G	HGMD
F193L	HGMD	F351S	HGMD	L69P	HGMD	S620G	HGMD	C981F*	HGMD	R1860S	HGMD
R195P	HGMD	L353P	ClinVar, HGMD	H70R	HGMD	S621R	HGMD	P1021S*	HGMD	A1870T	HGMD
K196T	HGMD	K354R	HGMD	H70N	HGMD	S621N	HGMD	D1166N*	HGMD	R1897W*	HGMD
P197S	HGMD	Q357R	HGMD	G71E	HGMD	T623I	HGMD	R1175C*	HGMD	E1901Q*	HGMD
I198V	ClinVar, HGMD	R360M	HGMD	G71R	ClinVar, HGMD	V625A	HGMD	P1177L*	HGMD	A1949S*	HGMD
S199A	HGMD	R360T	HGMD	G71W	HGMD	V625E	HGMD	Y1199S*	HGMD	E1954K*	HGMD
I200N	HGMD	K362R	HGMD	P72L	ClinVar, HGMD	G626A	HGMD	Y1241S	HGMD	Y1977N*	HGMD
D202G	ClinVar	H363N	ClinVar, HGMD	P72T	HGMD	G626D	HGMD	I1278N	HGMD	L1988R*	HGMD
D202H	HGMD	N365H	HGMD	T74P	HGMD	G626S	HGMD	N1325S	ClinVar, HGMD	R1991Q*	HGMD
L203P	HGMD	R366Q	ClinVar	T74R	ClinVar, HGMD	G626V	HGMD	A1326S	ClinVar, HGMD	F2004V*	HGMD
I204M	HGMD	R366P	HGMD	A78V	HGMD	F627I	HGMD				
I204F	HGMD	R366W	HGMD	A78T	HGMD	F627L	HGMD				
V205M	ClinVar, HGMD	Q367H	HGMD	A80P	HGMD	G628R	HGMD				
S209F	HGMD	A371T	HGMD	A85P	HGMD	G628D	HGMD				
K218E	HGMD	A372D	HGMD	A85V	HGMD	G628S	ClinVar, HGMD				
T224M	HGMD	S373P	HGMD	L86R	HGMD	G628V	HGMD				
S225L	HGMD	W379G	HGMD	L86P	HGMD	G628A	HGMD				
A226V	HGMD	W379S	HGMD	A89V	HGMD	N629D	ClinVar, HGMD				
I227L	HGMD	R380G	HGMD	E90K	HGMD	N629I	HGMD				
G229D	HGMD	R380S	HGMD	V94M	HGMD	N629K	HGMD				
R231C	HGMD	E385K	HGMD	E95G	HGMD	N629S	ClinVar, HGMD				
R231H	ClinVar, HGMD	S389P	HGMD	I96T	HGMD	N629T	HGMD				
I235N	ClinVar, HGMD	S389Y	HGMD	F98S	HGMD	V630A	HGMD				
L236R	HGMD	T391I	HGMD	Y99S	HGMD	V630L	HGMD				
L236P	HGMD	W392R	HGMD	R100Q	ClinVar, HGMD	S631A	HGMD				
L239P	HGMD	Y395S	ClinVar	R100W	HGMD	P632A	HGMD				
V241G	HGMD	K422T*	HGMD	K101E	HGMD	P632S	HGMD				
D242N	HGMD	T444M*	HGMD	D102A	HGMD	N633D	HGMD				
D242Y	HGMD	D446E*	HGMD	D102H	HGMD	N633I	HGMD				
R243C	ClinVar, HGMD	H455Y*	HGMD	D102V	HGMD	N633K	HGMD				
R243P	ClinVar, HGMD	R511W	HGMD	F106L	HGMD	N633S	HGMD				
G245V	HGMD	T513S	HGMD	F106Y	HGMD	T634A	HGMD				
W248C	HGMD	I517T	HGMD	C108R	HGMD	T634I	HGMD				
L250H	HGMD	M520R	HGMD	C108Y	HGMD	N635D	HGMD				
L250P	HGMD	Y522S	HGMD	L109R	HGMD	N635I	HGMD				
L251Q	HGMD	V524G	HGMD	L109P	HGMD	N635K	HGMD				
L251P	ClinVar, HGMD	A525T	HGMD	D111V	HGMD	E637D	HGMD				
G252D	HGMD	A525V	HGMD	P114S	HGMD	E637G	HGMD				
S253C	ClinVar	R539W	ClinVar, HGMD	M124R	HGMD	E637K	ClinVar, HGMD				
V254M	ClinVar, HGMD	E543K	HGMD	M124T	HGMD	K638N	HGMD				
V254L	HGMD	S546L	HGMD	F129I	HGMD	K638E	HGMD				
H258N	HGMD	Q547R	HGMD	D219V*	ClinVar	F640L	ClinVar, HGMD				
H258P	HGMD	G548D	HGMD	P334L*	ClinVar	F640V	HGMD				
H258R	ClinVar, HGMD	V554A	HGMD	I400N	HGMD	V644L	HGMD				
R259C	ClinVar, HGMD	R555C	ClinVar, HGMD	W410S	HGMD	V644F	HGMD				
R259L	ClinVar, HGMD	R555H	HGMD	L413P	HGMD	M645L	HGMD				
R259H	HGMD	R555S	HGMD	Y420C	HGMD	M645V	ClinVar, HGMD				
E261Q	HGMD	K557E	HGMD	A422D	HGMD	G648S	HGMD				
E261K	HGMD	R561G	HGMD	A422T	HGMD	S649P	HGMD				
E261V	HGMD	R562M	HGMD	P426H	HGMD	M651R	HGMD				
L262V	HGMD	R562S	HGMD	Y427C	HGMD	S654G	HGMD				
T265I	ClinVar, HGMD	L563P	HGMD	Y427H	HGMD	F656C	HGMD				
L266P	HGMD	S566F	HGMD	Y427S	HGMD	F656L	HGMD				
G269S	ClinVar, HGMD	S566P	HGMD	S428L	HGMD	G657R	HGMD				
G269D	ClinVar, HGMD	I567F	HGMD	S428P	HGMD	G657C	HGMD				
G272D	ClinVar	I567S	HGMD	A429P	HGMD	G657S	ClinVar, HGMD				
G272V	HGMD	I567T	HGMD	P451L	HGMD	I662T	HGMD				
L273F	ClinVar, HGMD	G568A	ClinVar, HGMD	D456Y	HGMD	R685H	HGMD				
L273R	HGMD	G568R	HGMD	L457P	HGMD	H687Y	HGMD				
F275S	HGMD	K569E*	HGMD	D460Y	HGMD	R694H	HGMD				
S277L	ClinVar, HGMD	S571L*	HGMD	F463L	HGMD	R696P	HGMD				
S277P	HGMD	F573L*	HGMD	D466Y	HGMD	S706C	HGMD				
S277W	HGMD	R583C*	HGMD	N470D	ClinVar, HGMD	A715V	HGMD				
Y278H	HGMD	R583G*	HGMD	T473N	HGMD	P721L	HGMD				
Y281C	HGMD	N586D	HGMD	T473P	HGMD	I728F	HGMD				
L282P	HGMD	N586S	HGMD	T474I	ClinVar, HGMD	R744P	HGMD				
A283T	HGMD	T587M	ClinVar, HGMD	Y475C	HGMD	R752Q	HGMD				
A302T	HGMD	G589D	ClinVar, HGMD	E480V	HGMD	R752W	HGMD				
A302V	HGMD	T587R	HGMD	I489F	HGMD	A753S	HGMD				
L303P	HGMD	A590T	HGMD	A490P	HGMD	K757N	HGMD				
W304R	HGMD	R591C	HGMD	A490T	ClinVar, HGMD	D767Y	HGMD				
W305R	HGMD	R591H	ClinVar, HGMD	H492Y	HGMD	V770A	HGMD				
W305L	ClinVar, HGMD	R591L	HGMD	Y493C	HGMD	D774Y	HGMD				
G306R	ClinVar, HGMD	R594Q	HGMD	Y493F	HGMD	G785A	HGMD				
G306V	HGMD	E596K	HGMD	Y493S	HGMD	G785V	HGMD				
V308D	HGMD	L602P	HGMD	W497L	HGMD	G785D	HGMD				
T309R	HGMD	I609N	HGMD	D501N	HGMD	E788D	HGMD				
T309I	HGMD	D611Y	HGMD	D501G	ClinVar, HGMD	E788K	HGMD				
V310I	HGMD	G635R*	HGMD	D501H	HGMD	V795I	HGMD				
T311A	HGMD			G522R	HGMD	G800A	ClinVar				
				K525N	HGMD	G800E	HGMD				
				R528P	HGMD	G800W	HGMD				
				R531Q	HGMD	M801I	ClinVar				
				R534C	ClinVar, HGMD	D803Y	HGMD				
				R534L	HGMD	F805C	HGMD				
				R537W	HGMD	F805S	ClinVar, HGMD				
				L552S	ClinVar, HGMD	G806E	ClinVar, HGMD				
				E544A	HGMD	P815L	HGMD				
				A558E	HGMD	G816V	HGMD				
				A558P	ClinVar, HGMD	S818L	HGMD				
				L559H	HGMD	S818P	HGMD				
				A561P	ClinVar, HGMD	S818W	HGMD				
				A561T	ClinVar, HGMD	G820E	HGMD				
				A561V	HGMD	G820R	ClinVar, HGMD				
				H562R	HGMD	V822I	ClinVar				
				W563C	HGMD	V822L	HGMD				
				W563G	HGMD	V822M	HGMD				
				L564P	HGMD	R823W	HGMD				
				A565T	HGMD	T826I	ClinVar, HGMD				
				C566F	HGMD	R835W	HGMD				
				C566S	HGMD	D837N	HGMD				
				W568R	HGMD	D837G	HGMD				
				W568C	HGMD	D837H	HGMD				
				Y569C	HGMD	D837Y	HGMD				
				Y569H	HGMD	V841L	HGMD				
				I571L	HGMD	P846T	HGMD				
				I571M	HGMD	I858T	HGMD				
				G572D	HGMD	N861H	HGMD				
				G572C	HGMD	N861I	ClinVar, HGMD				

Curated mutations classified as pathogenic in the ClinVar (Harrison et al., 2016) and HGMD (Stenson et al., 2012) databases. Mutations were cross-referenced to remove any mutations with conflicting classifications. Asterisked mutations are not mapped onto the channel structures presented in **Figures 9**–**11** because those positions are not resolved in the final models. The Excel file for this table can be found as “**Table 1**” in the **Supplementary Material**.

#### KCNQ1

KCNQ1 mutations studied to date are highly varied in molecular effects, with the potential to induce defects in channel stability, trafficking, electrophysiology, or all three (Chen et al., 2011; Heijman et al., 2012; Wu et al., 2016; Bohnen et al., 2017). So far, a strong disposition for either expression or functional defects has not been elucidated. However, analysis of mutation sites points to regions of increased pathological risk and provides hints regarding prevalent mechanisms.

LQT1-associated mutations are found in every domain of the protein (**Figure 9A**), particularly in the transmembrane channel domain (Shimizu et al., 2004; Moss et al., 2007; Kapa et al., 2009). Mutations in the SF, (**Figure 9A**, inset), VSD (**Figure 9B**), and the S4–S5 linker (**Figure 9C**) are especially prominent.

Mutations in and around the SF (**Figure 9A**, inset) point to a potential ion permeation defect, as conformational changes may prevent potassium ion permeation and thus decrease the effective I_Ks_ current (Choveau and Shapiro, 2012), as noted in several SF mutations (Ikrar et al., 2008; Thomas et al., 2010; Burgess et al., 2012; Chen et al., 2019). Molecular dynamics simulations of analogous mutations in KcsA indicated that mutations in the SF-interacting residues (including T322M, T322A, or G325R) may disrupt the organization of backbone atoms in the SF, abolishing K^+^ ion coordination and producing a dominant-negative effect on channel current (Burgess et al., 2012).

VSD mutations are more common in KCNQ1 compared to hERG and SCN5A (see **Figures 9B**, **10B**, and **11A**), indicating that the VSD is more important in LQT1. Analysis of LQTS mutations located in the VSD (Huang et al., 2018; Vanoye et al., 2018) indicate that loss of channel function was most commonly a consequence of mutation-induced destabilization of the channel, resulting in mistrafficking and lower surface expression. These results strongly correlated with Rosetta energy calculations of VSD mutants (Kuenze et al., 2019), supporting the importance of VSD destabilization as a LQT1 mechanism. A number of the deleterious mutations were found in the S0 helix and S0-contacting regions of the VSD. Among the 50 VSD mutations studied, the S0 mutations had the largest energetic destabilizations consistent with the large number of contacts formed between S0 and other regions of the VSD. (Huang et al., 2018; Kuenze et al., 2019). However, these studies also identified variants that did not exhibit folding and trafficking defects, but were either non-functional (low conductance) or dysfunctional, with modified gating, again pointing to a complex spectrum of LQT1 disease mechanisms.

Mutations in the KCNQ1 S4–S5 linker and its contacting regions (**Figure 9C**) have the potential to perturb VSD-PD coupling and channel gating. Such effects have been demonstrated for a number of LQTS-associated mutations in the S4–S5 linker and the cytoplasmic end of S6, particularly for R243C, W248F/R, and Q357R, producing positive shifts in the voltage dependence of activation or inactivation and and/or decreased current amplitude (Franqueza et al., 1999; Boulet et al., 2006; Mousavi Nik et al., 2015). Because the S4–S5 linker is involved in both VSD/PD interactions and in binding of PIP_2_ (Eckey et al., 2014; Sun and Mackinnon, 2017) and KCNE1 (Kang et al., 2008; Xu et al., 2013; Cui, 2016), mutations in this region may affect gating either by weakening VSD-PD coupling or by hindering modulation by auxiliary molecules. Some LQT1 variant-induced changes in PIP_2_ binding at the S4–S5 linker have been noted (including R243H, R539W, and R555C) (Park et al., 2005). Due to the complex nature of electromechanical coupling in KCNQ1 and the requirement for auxiliary subunits, a detailed structural understanding of mutation effects in this region is particularly challenging. However, the human KCNQ1, KCNQ1-KCNE3, and KCNQ1-KCNE3/PIP_2_ channel structures (Sun and Mackinnon, 2020) may provide guidance in experimental design to elucidate LQT1-associated coupling defects in this region.

While the majority of LQT1 mutations map to the transmembrane domain, mutations are also found in the CTD, both in the HA/HB helix bundle, where CaM binds, and in the HC and HD coiled-coil domains (**Figure 9A**). Mutations in HC and HD are particularly prevalent in sites of hydrophobic and electrostatic interactions that stabilize the coil, hinting at channel assembly defects (**Figure 9D**). Defects in CaM binding and tetramerization cause some forms of LQT1, as demonstrated for several mutations (Kanki et al., 2004; Ghosh S, 2006; Schwake et al., 2006; Wiener et al., 2008).

#### hERG

Loss-of-function mutations in hERG may lead to folding/trafficking defects, gating defects, or both (Anderson et al., 2014; Smith et al., 2016; Bohnen et al., 2017). However, as many as 90% of hERG loss-of-function mutations appear to cause trafficking defects, independent of which domain is mutated (Smith et al., 2016). As in KCNQ1, pore domain mutations in hERG demonstrated stronger dominant-negative effects in hERG than other regions, hinting that mutations in this region are more severe (Anderson et al., 2014).

Although hERG mutations span the entire protein, they are prominent in the SF (**Figure 10A**, inset), the extracellular VSD/PD interface between S1 and S5 (**Figure 10C**), and in the PAS and CNBHD cytoplasmic domains (**Figure 10D**). Given the inherent instability of the hERG SF (Fan et al., 1999; Stansfeld et al., 2008; Vandenberg et al., 2012) and the functional necessity of the SF for fast inactivation (Herzberg et al., 1998; Hoshi and Armstrong, 2013), mutations may act by both affecting channel stability and inactivation. Inactivation defects due to LQT2-associated pore helix mutations have been identified (Zhao et al., 2009; Poulsen et al., 2015). However, mutations in LQT2 more commonly cause misfolding and/or trafficking defects. Potassium ions appear to stabilize the mature hERG channel (possibly by interacting with the SF) to promote trafficking (Wang et al., 2009; Apaja et al., 2013), with intracellular K^+^ depletion resulting in ER retention. G601S mimicked this phenotype (Apaja et al., 2013). G601S, a mutation in the pore loop near the SF, mimicked also impedes trafficking through enhanced chaperone association in the ER (Ficker et al., 2003), supporting the idea of SF destabilization as a cause for channel mistrafficking. A detailed understanding of mutations in the SF region requires both intensive structural and cellular analysis to uncover not only protein conformational changes upon mutation, but also cellular quality control responses to these changes.

The VSD of hERG has fewer pathogenic mutations than that of KCNQ1 (**Figure 10B**), perhaps an indication that the hERG VSD has a higher folding stability. Indeed, when a set of hERG VSD mutations were assessed for membrane insertion efficiency, the majority of mutations were predicted to have no change in the free energy of insertion (Anderson et al., 2014). However, there are a surprising number of disease mutations at the extracellular interface between S1 and S5, specifically at the A422, P426, Y427, S428, H562, W563, C566, and Y569 positions (**Figures 10B, C**). This interface has not been studied extensively. However, we speculate that it may be important to channel stability by providing an additional contact surface between the VSD and the PD distinct from the S4–S5 linker. Destabilization of this interface may therefore lead to mistrafficking. Mutation Indeed, A422T results in ER retention but was rescued with a pharmacological chaperone (Guo et al., 2012). Other mutations in S1 alter gating properties in addition to trafficking (Anderson et al., 2014; Phan et al., 2017), indicating that gating properties may also be affected. Further studies are needed to determine the impact of mutations in this interface on folding, trafficking, and gating properties of hERG.

A number of mutations are found in the PAS and CNBHD, particularly in the PAS and at the interface between the two domains (**Figures 10A, D**). The structural stability of both domains impacts trafficking (Akhavan et al., 2005; Harley et al., 2012; Ke et al., 2013; Ke et al., 2014), and several trafficking-defective mutants have been characterized at the PAS/CNBHD interface (F29L, I31S, I42N, Y43C, R56Q, M124R), suggesting that occlusion of a PAS hydrophobic patch from the cytoplasm through interaction with the CNBHD is critical for proper trafficking (Ke et al., 2013). Trafficking defects due to destabilization of the PAS, the CNBHD, or their interaction may thus be a prominent mechanism in LQT2. The direct interaction of these two domains has also been implicated in slow deactivation of the channel (Ng et al., 2014; Kume et al., 2018), as deactivation defects have been noted in channels with mutations at this interface (Anderson et al., 2014). Deactivation defects may be a secondary source of LQT2. Detailed structural analysis of mutation-induced destabilization of the PAS and the CNBHD as well as studies of cellular responses to PAS/CNBHD destabilization would be highly informative to our understanding of LQT2. Indeed, similar studies have already revealed differential cellular responses to LQT2 PAS mutations, depending on the severity of PAS destabilization (Foo et al., 2019).

#### SCN5A

LQT3 mutations induce channel gain-of-function in SCN5A (Wilde and Amin, 2018; Skinner et al., 2019). Accordingly, mutations that interfere with SCN5A folding and trafficking do not cause LQT3 (Bohnen et al., 2017; Wilde and Amin, 2018). The primary mechanism leading to LQT3 is rather impairment of fast inactivation (Bohnen et al., 2017; Wilde and Amin, 2018). The results of our analysis are consistent with this notion. The site where the S5 and S6 helices of repeats III and IV cross, in the binding pocket for the IFM motif, contains the majority of the mutations curated in this study (**Figures 11A, C**). These mutations may impact interaction of the IFM motif with other channel structures, hindering fast inactivation and generating a persistent sodium current. For instance, A1326 (A1328 in rSCN5A) and G1329 (G1331 in rSCN5A) make critical backbone interactions to help form a tight hydrophobic pocket for IFM interactions (Jiang et al., 2020). Mutations A1326P and G1329S (**Table 1**) may thus compromise the stability of this pocket, hindering inactivation. Additionally, S6 mutations surrounding this putative inactivation gate also map to the constriction site of the channel pore, with additional mutations in S6_I_ and S6_II_ (**Figure 11D**). It has been proposed that binding of the IFM motif causes a rotation in the S6 helices that closes the pore (Yan et al., 2017b). Therefore mutations in S6 may also alter inactivation by preventing helix rotation. Indeed, residues important to defining the constriction site of the Na_v_1.7 pore cause LQTS upon mutation in SCN5A (L398 and Y1755) (Shen et al., 2019). A few mutations also map to the CTD of SCN5A (**Figure 11B**), which could alter interactions with the III/IV linker and hinder fast inactivation.

The recent structure of the rat homolog of SCN5A (Jiang et al., 2020) suggests another gain-of-function mechanism, namely that VSD mutant R225P could promote current leak through the VSD (gating pore current). Experimental evidence supporting this mechanism exists for other SCN5A (Moreau et al., 2015) and Na_V_1.4 (Sokolov et al., 2007) VSD mutations.

## Structural Basis for Channel Pharmacology

Pharmacological management of LQTS aims to minimize symptomatic arrhythmia and prevent life-threatening cardiac events. According to consensus guidelines (Zipes et al., 2006; Priori et al., 2013), treatment with β-blockers is beneficial and effective in the case of LQTS diagnosis, but should be considered only after additional diagnostics for carriers of LQTS mutations. β-blockers work by inhibiting β-adrenergic receptors, consequently slowing heart rate and preventing arrhythmias triggered by activation of the sympathetic nervous system (Bohnen et al., 2017). However, the efficacy of β-blockers is strongly genotype- and trigger-specific (Moss et al., 2000; Schwartz et al., 2001; Bohnen et al., 2017). β-blockers reduce the risk of exercise-induced arrhythmia by 78% and 71% in patients with LQT1 and LQT2, respectively, but neither are protective against cardiac events triggered by emotional arousal or during rest (Kim et al., 2010; Goldenberg et al., 2012). Patients with LQT3 tend to experience the highest breakthrough rate for cardiac events (10–15%) with β-blocker treatment (Giudicessi and Ackerman, 2013). Use of local anesthetic-type anti-arrhythmic agents (e.g. mexiletine) for clinical treatment of LQT3, however, can be accompanied by undesirable side effects (Moreno et al., 2011). The need for improved LQTS pharmacotherapy is further supported by the difficulties surrounding hERG modulation, as several clinically-used drugs can cause drug-induced LQTS by blocking hERG and suppressing I_Kr_ (Vandenberg et al., 2012; Kalyaanamoorthy and Barakat, 2018). This section summarizes some known pharmacological agents and the modes of interaction with KCNQ1, hERG, and SCN5A. Special focus is dedicated toward unique structural features of hERG that have been implicated in the promiscuity of this channel for pro-arrhythmic drugs.

### KCNQ1

KCNQ1 channels are targeted by several non-selective anti-arrhythmic drugs such as quinidine (Balser et al., 1991), amiodarone (Balser et al., 1991; Kamiya et al., 2001), azimilide (Busch et al., 1997), and clofilium (Yang et al., 1997). More specific KCNQ1 blockers include chromanol 293B (Bosch et al., 1998), and the benzodiazepine L-735821 (Lengyel et al., 2001). The binding site of L-735821 has been located to the SF and S6 (Seebohm et al., 2003a).

Benzodiazepine R-L3 was the first KCNQ1 activator to be described. The compound acts to slow the deactivation rate and causes a hyperpolarizing shift in the voltage-dependence of channel activation (Salata et al., 1998). R-L3 appears to bind residues on S5 and S6 lining the membrane-exposed surface of the PD (Seebohm et al., 2003b) rather than to the inner channel cavity. Drug binding outside of the PD cavity was also reported for quinidine (Yang et al., 2013), which occupies a pocket between S6 and the S4–S5 linker and is postulated to elicit an allosteric channel blocking mechanism. More recently, ML277 was identified as a potent KCNQ1 agonist (Mattmann et al., 2012). ML277 has been proposed to bind to a side pocket surrounded by the S2–S3 and S4–S5 linkers on the intracellular side and helices S4 and S6 on the intramembrane lateral side (Xu et al., 2015). Binding of ML277 may selectively alter VSD/PD coupling to stabilize the AO state relative to the IO state (Hou et al., 2019).

### hERG

**Figure 12:**
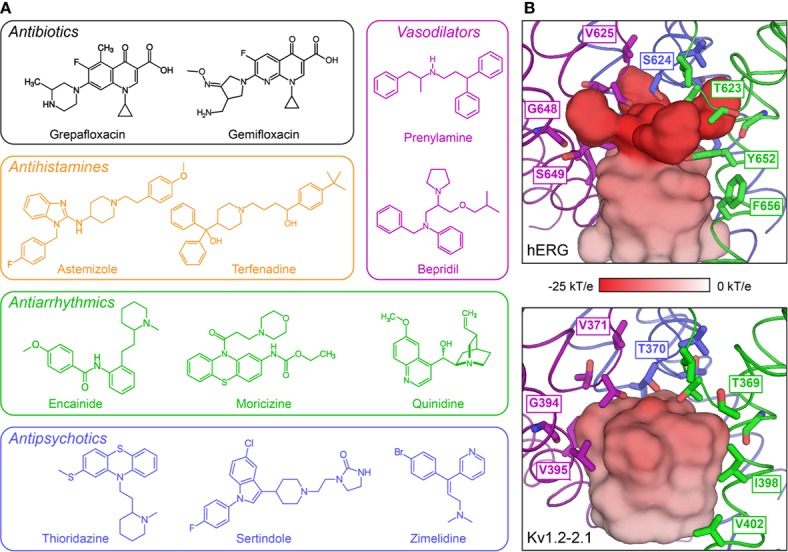
Structural features of the drug-binding cavity of the hERG channel pore. **(A)** Examples of drugs discontinued by the US FDA because of side effects related to hERG inhibition (adapted from **Table 1** in (Kalyaanamoorthy and Barakat, 2018)). **(B)** Internal molecular surface of the central cavity below the SF in hERG (PDB: 5VA1)(Wang and Mackinnon, 2017) (top) and the K_V_1.2–2.1 chimeric channel (PDB: 2R9R)(Long et al., 2007) (bottom). The surface is colored by the electrostatic potential calculated with the APBS tool in PyMOL (The PyMOL Molecular Graphics System, Version 2.2 Schrödinger, LLC.). Residues related to drug binding by hERG and the corresponding residues in the K_V_1.2–2.1 structure are shown as sticks. This panel is inspired by **Figure 5** in (Wang and Mackinnon, 2017).

hERG channels can be blocked by a wide spectrum of compounds, leading to drug-induced LQTS (Cubeddu, 2016). The list of drugs that inhibit hERG includes, among others, antibiotics (grepafloxacin (Bischoff et al., 2000)), antihistamines (astemizole (Zhou et al., 1999)), anti-arrhythmics (quinidine (Roden et al., 1986), dofetilide (Jurkiewicz and Sanguinetti, 1993)), antipsychotics (sertindole (Rampe et al., 1998)), and gastroprokinetic agents (cisapride (Vitola et al., 1998)) (**Figure 12A**). Several prescription drugs have been withdrawn from the market because of cardiotoxic side effects triggered by off-target interaction with hERG (Kalyaanamoorthy and Barakat, 2018). In fact, an estimated 15% of drugs still on the market may cause QT phase prolongation, and 60% of drugs in development show hERG liability (Vandenberg et al., 2017). It is now required by the US FDA that all compounds considered for advancement to clinical trials be tested for their impact on hERG channel function as part of preclinical toxicity assessments (Center for Drug Evaluation and Research, 2005a; Center for Drug Evaluation and Research, I.C.O.H., 2005b).

Considerable progress has been made in understanding the basic features of drug binding to hERG, and a plausible mechanism for promiscuity of drug binding has emerged. Pro-arrhythmic drugs commonly bind to the central inner cavity of the PD in hERG. Two pore-lining aromatic residues in S6 (Y652 and F656) are the most crucial for drug binding (Mitcheson et al., 2000), although additional residues at the end of the pore helix (T623, S624, and V625) contribute to binding for some drugs (Mitcheson et al., 2000), and mutations at F557 in the S5 helix can also affect binding (Saxena et al., 2016). The side chains of the S6 and pore helix residues point towards a putative drug binding site located in the central cavity below the SF. This cavity is slightly narrower in hERG than in the Shaker family K_V_1.2–2.1 chimeric channel (Long et al., 2007). As a consequence, there is a greater negative electrostatic potential in this region (Wang and Mackinnon, 2017), which could enhance drug binding capabilities (**Figure 12B**). In addition, four lateral hydrophobic pockets, which are not observed in the K_V_1.2–2.1 structure, extend outwards from that central constriction site, generating additional space to trap chemical moieties (Wang and Mackinnon, 2017).

Quantitative structure-activity relationship studies have identified a pharmacophore for drugs that bind to hERG (Cavalli et al., 2002). The pharmacophore consists of three centers of mass (usually aromatic rings) and an amino group that together form a flattened tetrahedron. It has been suggested that the aromatic rings could fit into one or more lateral hydrophobic pockets in hERG and/or bind by pi-pi stacking or hydrophobic interactions with Y652 and F656 (Chen et al., 2002). Likewise, the greater negative electrostatic potential in the central cavity could explain why many drugs that bind hERG, including off-targets, contain a positive charge (Fernandez et al., 2004). Additionally, a number of docking models for drug-bound hERG, with and without this pharmacophore, are being generated (Wacker et al., 2017; Helliwell et al., 2018; Negami et al., 2019), making possible further delineation of drug binding modes within this pocket and the structural basis of their affinity for hERG.

The majority of high-affinity drugs that bind hERG off-target preferentially bind to the inactivated state rather the open state, with affinity differences ranging from 2- to 100-fold (Perrin et al., 2008). Interestingly, the related EAG1 channel (Whicher and Mackinnon, 2016) is less sensitive to inhibition by most of hERG-blocking drugs even though they share the same pore-lining aromatic residues. One major difference between hERG and EAG1 is that EAG1 channels undergo minimal inactivation (Garg et al., 2012) whereas hERG channels undergo rapid and complete inactivation (Vandenberg et al., 2012). The contribution of inactivation to high-affinity binding is further substantiated by the observation that inactivating EAG1–hERG chimeras, containing the upper half of the hERG PD, can bind drugs with almost hERG-like affinity (Herzberg et al., 1998). While cryo-EM structures have been determined for both hERG and EAG1 (Whicher and Mackinnon, 2016; Wang and Mackinnon, 2017), the structural basis of the functional and pharmacological differences requires higher resolution (Wang and Mackinnon, 2017).

### SCN5A

Limited progress has been made in the development of SCN5A-targeted treatment strategies. Classical non-selective sodium channel blockers like the Vaughan-Williams class I anti-arrhythmic drugs quinidine, lidocaine, and propafenone have only limited clinical applicability. Ranolazine is a more selective SCN5A blocker (Belardinelli et al., 2006; Zaza et al., 2008) and has been shown to attenuate action potential duration and reduce or prevent intracellular calcium overload in LQT3 models (Wu et al., 2004; Lindegger et al., 2009). Mexiletine is a potent inhibitor of SCN5A and may be beneficial for treatment of LQT3 (Mazzanti et al., 2016), but effectiveness may depend on the baseline QT prolongation of the mutant channel (Li and Zhang, 2018).

Despite the clinical relevance of SCN5A-targeting drugs, the molecular mechanisms of promising and potentially problematic drugs are not well understood at a structural level, hindering drug design. However, key residues for apparent drug binding have been suggested within the sodium channel pore domain. Specifically, mutations of two conserved aromatic residues in S6_IV_, F1760 and Y1767, (Ragsdale et al., 1994; Ragsdale et al., 1996) or other S6 residues in repeats I and III (Yarov-Yarovoy et al., 2001; Yarov-Yarovoy et al., 2002) may affect drug binding. Using fluorinated phenylalanine derivatives incorporated at F1760 (Pless et al., 2011a), a strong cation-pi interaction between F1760 and the protonated amine group in class Ib anti-arrhythmic drugs was observed, defining the molecular basis for inhibition of SCN5A by this group of drugs. Additionally, mutations within the SF region can affect apparent drug binding either by enhancing slow inactivation or forming an alternative access pathway (Sunami et al., 1997; Lee et al., 2001; Tsang et al., 2005). Interactions of anti-arrhythmic and local anesthetic drugs (lidocaine, QX-314, etidocaine, flecainide, and ranolazine) docked into an inactivated state model of SCN5A (Nguyen et al., 2019) revealed several key drug binding sites in the inner pore lumen that can simultaneously accommodate up to two drug molecules. Subsequent MD simulations suggested alternative access mechanisms for drugs into the SCN5A lumen – a hydrophilic pathway through the intracellular gate and a hydrophobic pathway through a membrane-exposed fenestration between repeats III and IV.

The recent structure of rat Na_v_1.5 (Jiang et al., 2020) has provided insight into the binding mode of the class Ic anti-arrhythmic drug flecainide, which has higher affinity for the open conformation of SCN5A (Ramos and O’leary M, 2004). Flecainide binds in the central cavity on the intracellular side of the SF. The piperidine ring of flecainide lies across the top of the central cavity with the positively-charged nitrogen pointing towards the exit of the SF and its hydrophobic edge extending towards the phenyl ring of F1762 (F1760 in human Na_v_1.5). The trifluoroethoxy tails of flecainide extend into fenestrations formed between the pore helices of repeats I and II as well as repeats II and III, through which the central cavity is accessible from the lipid bilayer. These fenestrations represent possible entry points for flecainide and similar class Ic drugs.

## Future Directions

The growing number of structural and functional studies of KCNQ1, hERG, and SCN5A have provided new insight into channel molecular physiology and channel dysfunction in congenital LQTS. This further motivates the study of fundamental biophysical mechanisms in ion channels. One of these mechanisms, electromechanical coupling, remains enigmatic due to a dearth of experimental resting state structures and robust methods to identify allosteric pathways, which cannot easily be discerned from ion channel structures resolved in a single conformation. However, interaction energy and MD dynamical network analysis in the Shaker K^+^ channel (Fernandez-Marino et al., 2018) has suggested that movements in the VSD and PD are linked by two distinct pathways – a canonical pathway through the S4–S5 linker and a hitherto unknown non-canonical pathway involving contacts at the interface between S4 and S5. Such work may provide a useful alternative approach to explore electromechanical coupling in detail.

Another major effort involves the experimental characterization of hundreds of previously uncharacterized variants of unknown significance (VUS) and LQTS mutations in KCNQ1, hERG, and SCN5A. Widespread use of whole genome and exome sequencing has provided a nearly complete catalog of common sequence variations in protein-coding genes (Lek et al., 2016), although rare sequence variants continue to be discovered. For many LQTS mutations, the molecular mechanisms responsible for impaired channel function remain poorly understood. Such mutations may cause potassium channel loss-of-function or dysfunction by promoting misfolding, mistrafficking, aggregation, or improper gating of the channel protein, as discussed above. Continued experimental testing will be required to complete functional annotation of KCNQ1, hERG, and SCN5A variants and to elucidate how these mutations lead to channel dysfunction. In this regard, new techniques such as high-throughput patch clamp recording (Vanoye et al., 2018; Toh et al., 2020), which allows rapid screening of ion channel variants found in patients, and the use of induced-pluripotent stem cell-derived cardiomyocytes (Moretti et al., 2010; Ma et al., 2013; Terrenoire et al., 2013; Jouni et al., 2015) developed from cells of healthy or mutation-carrying patients, represent helpful tools for large-scale examination of ion channel variants.

Finally, experimentally-informed bioinformatics and modeling approaches are being developed to predict variant pathogenicity (Kroncke et al., 2015; Li et al., 2017; Kroncke et al., 2019). These computational algorithms can help to decrypt newly discovered VUS for which there is not enough data to determine pathogenicity. Decrypting VUS can inform medical practice and has the potential to improve LQTS therapy, both for the prevention of cardiac events in susceptible patients and as a basis for avoiding unneeded treatment in healthy patients. With improved understanding of the molecular pathophysiology and the role of mutations associated with distinct LQTS subtypes, the ultimate hope is that identifying a patient’s genotype will lead to more specific treatment considerations and the delivery of optimal care.

## Author Contributions

KB and GK wrote the primary draft of this article, with edits by KB, GK, JM, CV, AG, and CS. KB and GK contributed equally to this manuscript.

## Funding

This work was supported by US NIH grant RO1 HL122010. GK was supported by a postdoctoral fellowship from the American Heart Association (18POST34080422) and KB was supported by NIH training grant T32 GM008320.

## Conflict of Interest

The authors declare that the research was conducted in the absence of any commercial or financial relationships that could be construed as a potential conflict of interest.

## Supplementary Material

Table 1 LQTS mutations in KCNQ1, hERG, and SCN5A.
